# Metabolic engineering for microbial production of sugar acids

**DOI:** 10.1186/s12896-025-00973-7

**Published:** 2025-05-13

**Authors:** Fatma Gizem Avci, Tim Prasun, Volker F. Wendisch

**Affiliations:** 1 Genetics of Prokaryotes, Faculty of Biology and Center for Biotechnology (CeBiTec), Bielefeld University, Bielefeld, Germany; 2 Department of Bioengineering, Faculty of Engineering and Natural Sciences, Üsküdar University, Istanbul, Türkiye

**Keywords:** Sugar acids, Metabolic engineering, Biocatalysis, Bioconversion, Fermentation, Microorganism

## Abstract

Carbohydrates including sugar acids are commonly used as carbon sources in microbial biotechnology. These sugar acids are themselves desirable and often overlooked targets for biobased production since they find applications in a broad range of industries, examples include food, construction, medical, textile, and polymer industries. Different stages of oxidation for natural sugar acids can be distinguished. Oxidation of the aldehyde group yields aldonic acids, oxidation of the primary hydroxy group leads to uronic acids, and both oxidations combined yield aldaric acids. While the chemical oxidation of sugars to their acid forms often is a one-pot reaction under harsh conditions, their biosynthesis is much more delicate. Bio-based production can involve enzymatic conversion, whole-cell biotransformation, and fermentation. Generally, the in vivo approaches are preferred because they are less resource-intensive than enzymatic conversion. Metabolic engineering plays a crucial role in optimizing microbial strains for efficient sugar acid production. Strategies include pathway engineering to overexpress key enzymes involved in sugar oxidation, deletion of competing pathways to enhance the precursor availability and eliminate the product consumption, cofactor balancing for efficient redox reactions, and transporter engineering to facilitate precursor import or sugar acid export. Synthetic biology tools, such as CRISPR-Cas and dynamic regulatory circuits, have further improved strain development by enabling precise genetic modifications and adaptive control of metabolic fluxes. The usage of plant biomass hydrolysates for bio-based production further adds to the environmental friendliness of the in vivo approaches. This review highlights the different approaches for the production of C5 and C6 sugar acids, their applications, and their catabolism in microbes.

## Background

Sugar acids are organic acids that are the oxidation products of mono- or oligosaccharides. Conventionally, these acids are produced by electrochemical or chemical oxidation. However, scaling up these processes industrially is challenging due to the use of costly or harmful catalysts and the formation of undesirable by-products, which complicate the downstream processing and reduce overall efficiency [1, 2].

Bio-based production methods offer significant advantages by replacing fossil fuels with renewable sources and lowering carbon emissions. Specifically, microorganisms are essential platforms for the bioproduction of different molecules. However, the limited performance of microbial strains and processes constrains the commercialization of microbe-derived compounds [3, 4].

Metabolic engineering methods have enabled efficient and sustainable production of a wide range of chemicals by microbial cell factories. Various genome-editing technologies, such as CRISPR-Cas systems and recombineering, have revolutionized strain improvement by enabling precise modifications in microbial genomes. Furthermore, the optimization of genetic elements—such as promoters, ribosome binding sites (RBSs), terminators, and regulatory RNA sequences—has enhanced gene expression control, thereby improving metabolic flux toward the biosynthesis of target molecules. Well-established metabolic engineering approaches include pathway rewiring, cofactor balancing, transporter engineering, and adaptive laboratory evolution (ALE) [4]. Most of these strategies have been successfully employed for the biosynthesis of several sugar acids such as D-gluconic acid, D-glucaric acid, and D-galacturonic acid, among others. In the current review, primarily focusing on microbial metabolic engineering, the production of C5 and C6 sugar acids using several methods was summarized. Additionally, the properties, classification, and industrial applications of the sugar acids, along with insights into their catabolic pathways and microbial utilization were provided. This review aims to offer a comprehensive understanding of the advancements and challenges in microbial sugar acid production, highlighting strategies for improving yield and scalability.

## Sugar acids: classification, properties and applications

### Structural classification

**Fig. 1:**
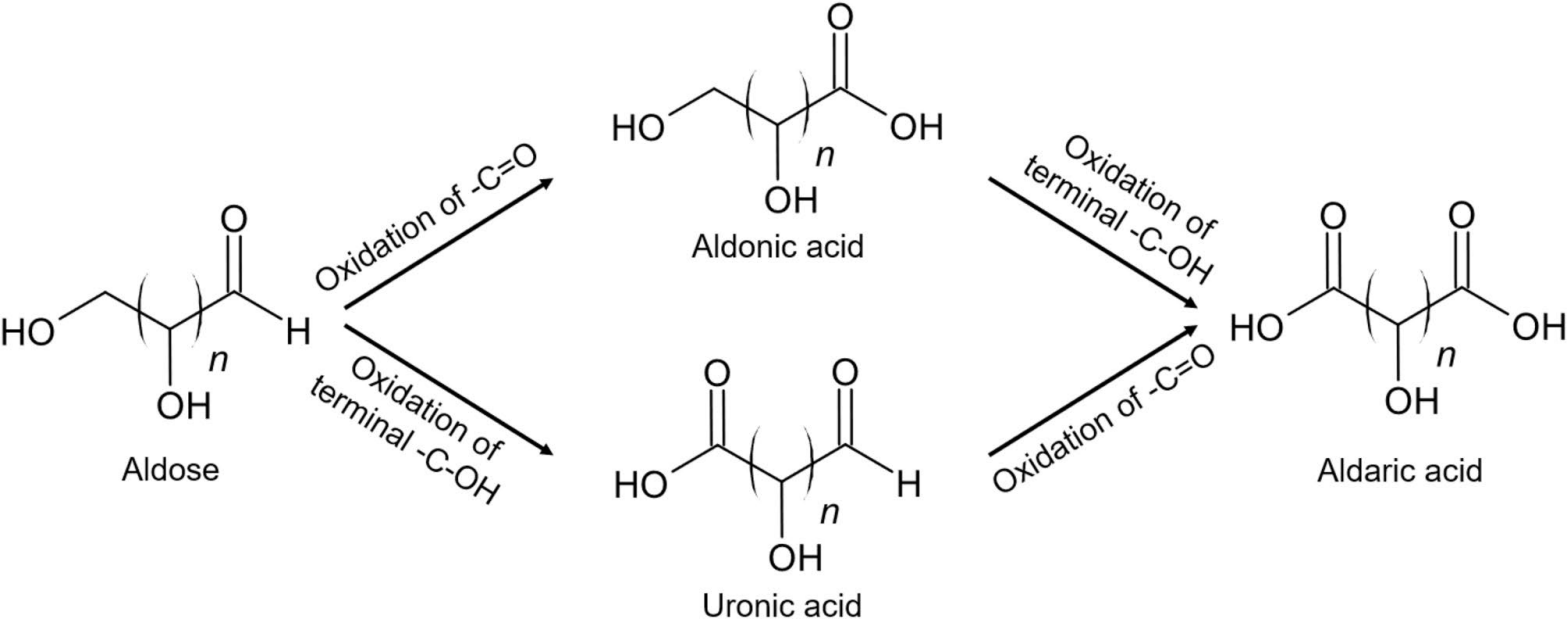
General structures of sugar acids that are oxidized from aldoses

Sugar acids are oxidized monosaccharides classified into four main classes: Aldonic, uronic, aldaric, and ulosonic acids. In aldonic acids, the terminal aldehyde group (R-CHO) of an aldose is oxidized to a carboxyl group (R-COOH). For uronic acids, the hydroxymethyl group (R-CH_2_OH) furthest from the carbonyl group is oxidized to a carboxyl group. Aldaric acids are obtained by oxidation of both the aldehyde and the terminal hydroxymethyl groups, forming dicarboxylic acids (Fig. 1). Ulosonic acids are polyhydroxy 2-oxoacids formed by the oxidation of a ketose’s terminal hydroxymethyl group. They occur rarely in nature, for example as lipopolysaccharide components of Gram-negative bacteria [5], and are therefore excluded in this review.

### General properties

Sugar acids share most structural properties with their non-oxidized carbohydrate counterparts. The geometry of the linear carbohydrate does not change during oxidation. Aldonic acids form lactones instead of cyclic hemiacetals and lose their reducing ability due to an already oxidized anomeric carbon. In contrast to aldonic acids, uronic acids can still circularize into cyclic hemiacetals. Since aldaric acids have no available carbonyl group, they cannot form cyclic acetals, some can however, circularize into lactones [6]. During oxidation, aldaric acids (e.g., galactaric acid) might gain a new symmetry plane and become optically inactive.

As polyhydroxy dicarboxylic acids, aldaric acids are desirable for chemical synthesis. They can be converted into non-hydroxylated dicarboxylic acids [7, 8], which serve as precursors in the bioplastics industry, forming polyamides (nylon) or polyesters through polymerization.

### Applications

Sugar acids often exist in a variety of structural and stereoisomeric forms, with oxidation enhancing their functionality and reactivity for numerous applications. The presence of hydroxyl and carboxylic acid groups makes sugar acids attractive for use in polymerization processes, contributing to biodegradable materials like polyamides, polyesters, and polyurethanes, which have potential uses in biomedical products and food packaging. Sugar acids are also valuable for use in the food, cosmetics, and pharmaceutical industries [2, 9, 10].

**Fig. 2:**
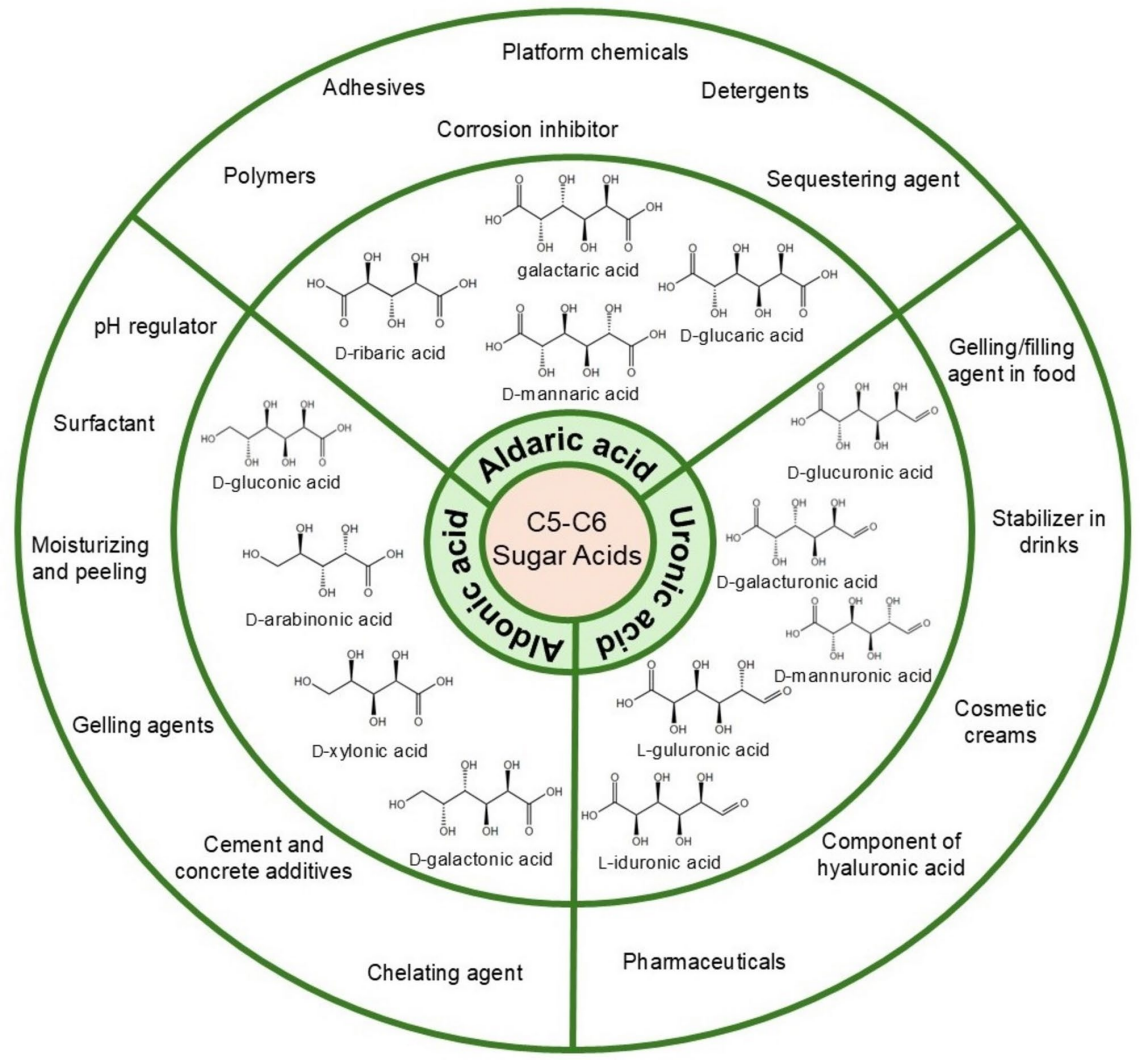
Applications of C5 and C6 sugar acids

The unique structures and properties of each class make the sugar acids favorable in several areas (Fig. 2). Aldonic acids and their derivatives serve as pH regulators and gelling agents [11, 12], moisturizing and peeling agents [13], preservatives for organ transplants [14], chelating agents [15, 16], surfactants [17], textile bleaching aid [18], and construction [19]. Aldaric acids, identified as key bio-based chemicals by the US Department of Energy [20, 21], are considered promising raw materials for adhesives [22], crosslinkers in hydrogels [23], metal complexation agents [24, 25], detergents [26], and corrosion inhibitors [27]. Uronic acids are highly valuable chemicals used especially in the food, pharmaceutical, and cosmetic industries functioning as gelling and filling agents in food [28], stabilizers in juices and milk-based drinks [29], a building block of hyaluronic acid [30], an important component of glycosaminoglycans such as heparin, heparan sulfate, and dermatan sulfate [31], and cosmetic ingredient in moisturizing and protective skin treatment creams [32]. Ulosonic acids play crucial roles in biological processes, particularly in antibiotic development and bacterial vaccine research [33].

## Cellular transport and catabolism of C5 and C6 sugar acids

### Transport proteins for sugar acids

The transport of sugar acids across the cell membrane of the overproducing microorganism is relevant for strain development by metabolic engineering. For bioconversion of one sugar acid into another, e.g., D-galacturonic acid to galactaric acid, transport engineering may conceptionally improve both the uptake of the substrate D-galacturonic acid and the export of the product galactaric acid. Therefore, we will discuss sugar acid transport mechanisms.

Sugar acids enter and leave cells by crossing the cytoplasmic membrane via transport proteins. Identification of specific transporters for C5-C6 sugar acids in microbial cells can be challenging, as detailed information is rarely available. However, some specific transporters of C5-C6 sugar acids have been characterized in several, mainly model, microorganisms.

#### Bacterial transport

**Fig. 3:**
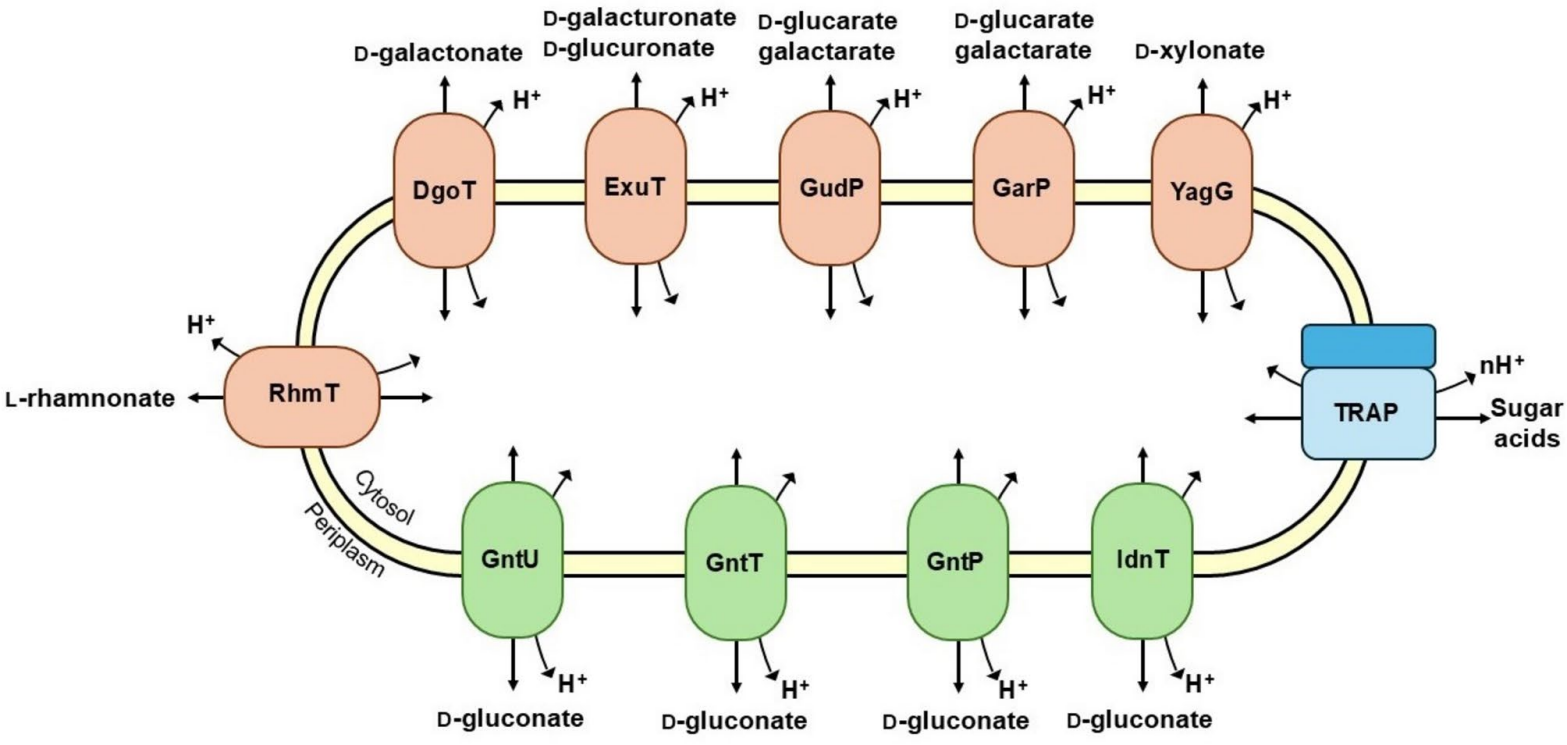
Sugar acid transporters in *E. coli*. The transporters in MFS are represented by orange, while green transporters belong to IT Superfamily. TRAP transporters (indicated in blue) can transport various sugar acids as identified by Vetting et al. [41]

*Escherichia coli* is one of the well-studied organisms for the sugar acid transporters. The sugar acid transporters that were characterized include DgoT (D-galactonic acid) [34], ExuT (D-galacturonic acid, D-glucuronic acid) [35], GarP and GudP (D-glucaric acid, galactaric acid) [36], GntU, GntP, GntT, and IdnT (D-gluconic acid) [37, 38], YagG (D-xylonic acid) [39], and RhmT (L-rhamnonic acid) [40] (Fig. 3). These transporters are mainly included in the Major Facilitator Superfamily (MFS) or Ion Transporter Superfamily (IT). Many microorganisms also have orthologous proteins for the transport of different sugar acids.

Solute binding proteins (SBPs) in transport systems facilitate the first step in a catabolic pathway by transporting metabolites across the cellular membrane. Transporter genes are often colocated or coregulated with genes encoding enzymes that metabolize the transported molecule. SBPs, found in the periplasm of Gram-negative bacteria or tethered to the outer membrane in Gram-positive bacteria, capture the initial reactant and deliver it to transmembrane components for cytosolic translocation [42, 43]. Three SBP-dependent transport systems have been identified: (1) Tripartite ATP-independent Periplasmic (TRAP) transporters, (2) ATP-Binding Cassette transporters, and (3) Tripartite tricarboxylate transporters. The TRAP system consists of a large transmembrane subunit (DctM with 12 helices), a small transmembrane subunit (DctQ with 4 helices), and an SBP (DctP, 320 residues) and transports by coupling to an electrochemical gradient, with a conserved arginine in the SBP ligand binding sites preferring organic acids [44]. These transporter systems are not previously associated with sugar transport, and in fact do not transport the sugars directly at all, rather requiring them to be converted in the periplasm to their respective sugar acid forms before transport through what appears to be a novel general sugar acid transporter [45]. Vetting et al. screened 158 SBPs against an 189-component library specifically tailored for this class of proteins. D + L-galacturonic acid, D-glucuronic acid, D-mannuronic acid, L-guluronic acid, D-taluronic acid, D-xylonic acid, D + L-ribonic acid, D-arabinonic acid, D-talonic acid, D-mannonic acid, D-gluconic acid, L-gulonic acid, D-allonic acid, D + L-galactonic acid, L-fuconic acid, and L-arabinaric acid were among the ligands determined for the TRAP SBPs [41].

CxaP (D-xylonic acid, D-fuconic acid, D-galactonic, and D-gluconic acid) [46], KguT (D-xylonic acid) [47], GntP (D-xylonic acid) [48], and UxuT (D-glucuronic acid) [49] are some other transporters identified in various bacteria.

#### Fungal transport

The Jen family is a member of the MFS and is associated with the plasma membrane transport of carboxylic acids in fungi. Ribas et al. [50] screened various yeast carboxylic acid transporters from the Jen family for their ability to transport sugar acids, including D-gluconic, D-glucaric, galactaric, D-xylaric, and D-xylonic acids. These transporters were functionally characterized in *Saccharomyces cerevisiae*. The results demonstrated that Jen permeases can transport most of these sugar acids with varying specificities. Specifically, D-glucaric acid is a substrate for the transporters ScJen1-S271Q and KlJen2, D-gluconic acid for CaJen2 and KlJen2, and D-xylaric acid and galactaric acid for CaJen2. A molecular docking approach with these transporters identified key residues involved in the substrate binding of these sugar acids. Specifically, the residues R188 in ScJen1, R122 in CaJen2, and R127 in KlJen2, all located in transmembrane segment II, play a major role in substrate binding. GAT-1 (D-galacturonic acid) [51], GatA (D-galacturonic acid) [52], and Ght3 (D-gluconic acid) [53] are also among the identified sugar acid transporters in several fungi.

#### Catabolism of sugar acids

One of the main metabolic engineering strategies to enhance product yields is to prevent the degradation/catabolism of the products. Sugar acids may be catabolized directly (such as D-gluconic acid which is phosphorylated upon uptake into the cell) or via other free sugar acids as intermediates (such as in the catabolism of D-galacturonic acid via D-tagaturonic acid and D-altronic acid in the Ashwell isomerase pathways). Therefore, we describe the catabolism of selected sugar acids in some detail to provide guidance for metabolic engineering.

#### Catabolism of D-xylonic acid

D-xylonic acid is an intermediate of the D-xylose catabolic pathway in some bacteria, e.g., *Caulobacter crescentus*. D-xylose is first oxidized to D-xylono-1,4-lactone by an NAD(P)^+^ dependent D-xylose dehydrogenase. This reaction has a 10-fold higher *k*_*cat*_ than the reverse reaction [54]. The lactone can hydrolyze spontaneously or via D-xylono-1,4-lactonase to form D-xylonic acid [55]. D-xylonic acid can then be dehydrated to 2-oxo-3-deoxy-D-xylonic acid catalyzed by a D-xylonate dehydratase [56], leading to two pathways: the Weimberg pathway, where it is further dehydrated by a dehydratase and then oxidized to 2-oxoglutarate by 2-oxoglutarate semialdehyde dehydrogenase [57], or the Dahms pathway, where it is cleaved into pyruvate and glycolaldehyde by a specific aldolase [58]. Glycolaldehyde can then be oxidized to glycolate through glycolaldehyde dehydrogenase activity or reduced to ethylene glycol catalyzed by a glycolaldehyde dehydrogenase.

#### Catabolism of hexonic acids

**Fig. 4:**
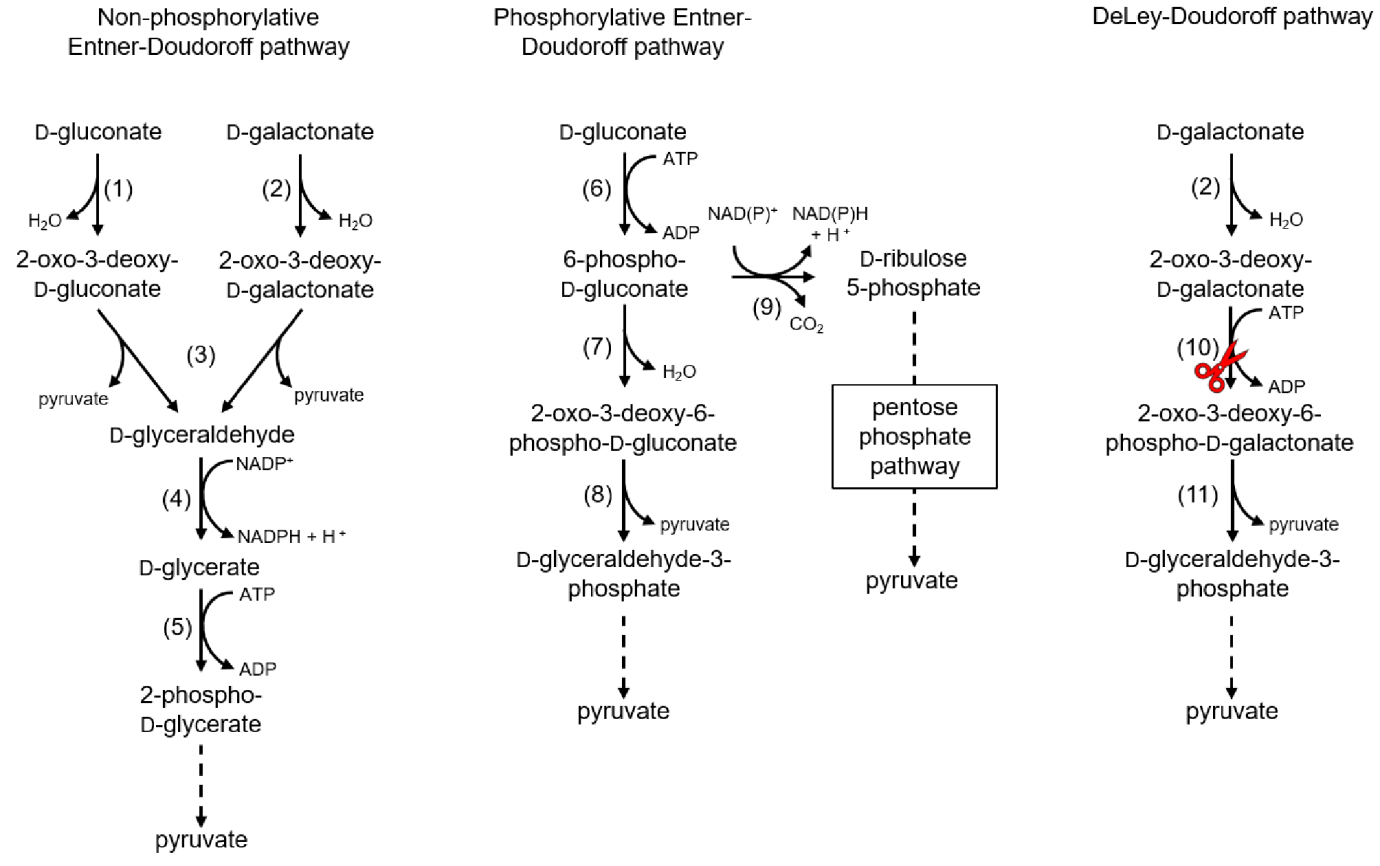
The catabolism pathways for D-gluconic acid and D-galactonic acid in bacteria. Enzymes: (1) D-gluconate dehydratase (2) D-galactonate dehydratase (3) 2-oxo-3-deoxy-D-gluconate aldolase (4) D-glyceraldehyde dehydrogenase (5) D-glycerate kinase (6) D-gluconate kinase (7) 6-phospho-D-gluconate dehydratase (8) 2-oxo-3-deoxy-6-phospho-D-gluconate aldolase (9) 6-phospho-D-gluconate dehydrogenase (10) 2-oxo-3-deoxy-D-galactonokinase (11) 2-oxo-3-deoxy-6-phospho-D-galactonate aldolase. Red scissors indicate genes that were deleted during metabolic engineering for the production of sugar acids.

Since D-gluconic acid and D-galactonic acid are epimers, many enzymes are allowed to process both, albeit with a lower affinity [59]. D-gluconic acid and D-galactonic acid can be utilized in the non-phosphorylative Entner-Doudoroff pathway [60]. Additionally, D-galactonic acid can be degraded in the DeLey-Doudoroff pathway [61], while D-gluconic acid can be utilized in the pentose phosphate pathway and the phosphorylative Entner-Doudoroff pathway [62] (Fig. 4).

In the non-phosphorylative Entner-Doudoroff pathway of thermophilic microorganisms, the D-gluconate or D-galactonate dehydratases dehydrate D-gluconic acid to 2-oxo-3-deoxy-D-gluconic acid or 2-oxo-3-deoxy-D-galactonic acid, respectively [59, 63, 64]. Subsequently, an aldolase cleaves both products into pyruvate and D-glyceraldehyde [65–67]. D-glyceraldehyde can then be oxidized to D-glyceric acid by the respective dehydrogenase and is then phosphorylated under ATP consumption to 2-phospho-D-glyceric acid, which can be further converted into pyruvate in the glycolysis [66].

D-gluconic acid can enter the phosphorylative Entner-Doudoroff pathway after phosphorylation to 6-phospho-D-gluconic acid by D-gluconate kinase [62, 68]. 6-Phospho-D-gluconic acid is dehydrated to 2-oxo-3-deoxy-6-phospho-D-gluconic acid by a 6-phospho-D-gluconate dehydratase [69]. The respective aldolase cleaves the product into D-glyceraldehyde-3-phosphate and pyruvate, both of which enter glycolysis [70]. After the initial phosphorylation, 6-phospho-D-gluconic acid can also be used in the pentose phosphate pathway by oxidative decarboxylation through a 6-phospho-D-gluconate dehydrogenase to D-ribulose 5-phosphate [71].

The DeLey-Doudoroff pathway [61] for D-galactonic acid utilization was identified in *E. coli* K-12 [72], mycobacteria [73], and archaea [59]. The initial step is shared with the non-phosphorylative Entner-Doudoroff pathway. The 2-oxo-3-deoxy-D-galactonic acid is then phosphorylated on its C6 position in a reaction catalyzed by a 2-oxo-3-deoxy-D-galactonokinase to 2-oxo-3-deoxy-6-phospho-D-galactonic acid. The product is then cleaved into D-glyceraldehyde-3-phosphate and pyruvate which can then enter the central metabolism [72].

#### Catabolism of hexuronic acids

The fungal D-galacturonic acid degradation is different from the bacterial catabolism. D-galacturonic acid is first reduced to L-galactonic acid by a NAD(P)H-dependent D-galacturonate reductase [74, 75]. Subsequent dehydration by L-galactonate dehydratase leads to 2-oxo-3-deoxy-L-gluconic acid [76]. A specific aldolase catalyzes the cleaving of this intermediate into pyruvate and L-glyceraldehyde, which is further reduced to D-glycerol through the activity of a L-glyceraldehyde reductase [77, 78].

**Fig. 5:**
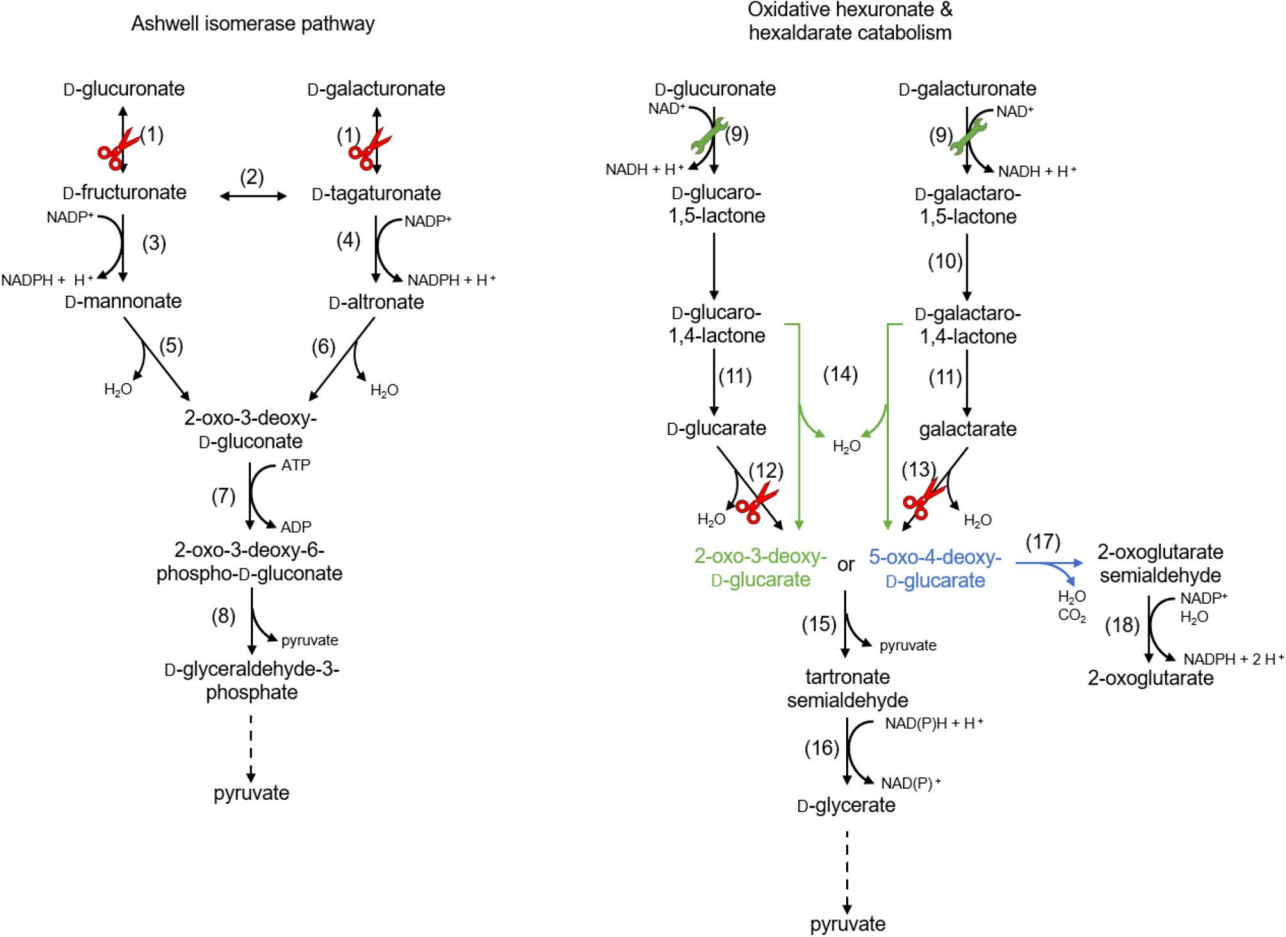
The catabolism pathways for D-glucuronic acid, D-galacturonic acid, D-glucaric acid, and galactaric acid in bacteria. Enzymes: (1) uronate aldose-ketose isomerase (2) D-tagaturonate/D-fructuronate epimerase (3) D-fructuronate reductase (4) D-tagaturonate reductase (5) D-mannonate dehydratase (6) D-altronate dehydratase (7) 2-oxo-3-deoxy-D-gluconate kinase (8) 2-oxo-3-deoxy-6-phospho-D-gluconate aldolase (9) uronate dehydrogenase (10) galactaro δ-isomerase (11) spontaneous or lactonase (12) D-glucarate dehydratase (13) galactarate dehydratase (14) D-galactarolactone isomerase (15) 5-oxo-4-deoxy-D-glucarate aldolase (16) tartronate semialdehyde reductase (17) 5-oxo-4-deoxy-D-glucarate dehydratase (18) 2-oxoglutarate semialdehyde dehydrogenase. Reaction arrows indicated in green point to 2-oxo-3-deoxy-D-glucarate/5-oxo-4-deoxy-D-glucarate. The arrow in blue indicates a reaction from 5-oxo-4-deoxy-D-glucarate only. Red scissors indicate genes that were deleted, and green wrenches indicate genes that were expressed during metabolic engineering for the production of sugar acids

A common pathway for the catabolism of D-glucuronic acid and D-galacturonic acid in bacteria is the Ashwell pathway (isomerase pathway) (Fig. 5). The genes for the responsible enzymes are encoded by the *uxaCBA* and *uxuAB* operons in *E. coli* [79, 80]. After uptake, the alduronic acids D-glucuronic acid and D-galacturonic acid are converted to their respective keturonic acids D-fructuronic acid and D-tagaturonic acid [81] by the uronate aldose-ketose isomerase UxaC [79, 82]. In the hyperthermophilic bacterium *Thermotoga maritima*, these two keturonic acids can be interconverted in a reaction catalyzed by a D-tagaturonic acid/D-fructuronic acid epimerase UxaE. Other microorganisms that possess an *uxaE* gene were identified [83]. In the next step, two enzymes with rather specific activities for either D-fructuronic acid or D-tagaturonic acid form D-mannonic acid by the reduction of the carbonyl group of D-fructuronic acid to a hydroxyl group through the activity of a NADH-dependent D-fructuronate reductase UxuB. The enzyme also accepts D-tagaturonic acid as a substrate with lower activity [84].

Analogously, a NAD(P)H-dependent D-tagaturonate reductase UxaB catalyzes the reduction of D-tagaturonic acid to yield D-altronic acid [82, 85, 86]. In the next step, the degradation pathways converge. D-mannonic acid and D-altronic acid can be dehydrated to 2-oxo-3-deoxy-D-gluconic acid by specific iron-dependent dehydratases, the D-mannonate dehydratase and the D-altronate dehydratase, respectively [79, 87, 88]. 2-Oxo-3-deoxy-D-gluconic acid can then be phosphorylated and utilized in the phosphorylative Entner-Doudoroff pathway, as described before for aldonic acid catabolism.

An alternative pathway used by i.a. *Agrobacterium tumefaciens* and *Pseudomonas syringae* is the oxidative D-galacturonic acid/D-glucuronic acid degradation [89]. D-galacturonic acid/D-glucuronic acid is first oxidized to D-galactaro/D-glucaro-1,5-lactone using an NAD^+^-dependent uronate dehydrogenase (Udh) [90]. The 1,5-lactone is then spontaneously converted to the more stable 1,4-lactone. A galactaro δ-isomerase is known [91]. Hydrolysis of the lactones to galactaric acid/D-glucaric acid occurs either spontaneously or is catalyzed by a lactonase [92]. The degradation of these aldaric acids is described in the next part.

#### Catabolism of hexaldaric acids

*E. coli* can utilize D-glucaric acid and galactaric acid as sole carbon sources [93, 94]. After uptake, specific dehydratases (GurD and GarD) dehydrate them to 2-oxo-3-deoxy-D-glucaric acid or 5-oxo-4-deoxy-D-glucaric acid, respectively (Fig. 5) [95, 96]. A specific aldolase catalyzes the cleavage into pyruvate and tartronate semialdehyde [95]. In the subsequent step, a tartronate semialdehyde reductase reduces tartronate semialdehyde to D-glyceric acid, which is oxidized and subsequently phosphorylated to the glycolysis intermediate 2-phospho-D-glyceric acid [96, 97]. Alternatively, 5-oxo-4-deoxy-D-glucaric acid can be dehydrated and decarboxylated to 2-oxoglutarate semialdehyde with subsequent oxidation to 2-oxoglutarate, as described before [98].

During the dehydration of D-glucaric acid and galactaric acid, another metabolite, 2-dehydro-3-deoxy-D-glucaric acid, can be synthesized [89, 92]. A D-galactaro-1,4-lactone isomerase can catalyze the reaction directly from D-galactaro-1,4-lactone and D-glucaro-1,4-lactone [92]. The same dehydratase that catalyzes the dehydration of 5-oxo-4-deoxy-D-glucaric acid can catalyze the reaction of 2-dehydro-3-deoxy-D-glucaric acid to 2-oxoglutarate semialdehyde.

## Chemo- and biocatalytic production of sugar acids

### Synthesis of sugar acids by chemical catalysis

Polysaccharides and lignocellulosics from plant biomass can be used in chemical synthesis. As opposed to petroleum-derived substrates, biomass contains more oxygen, e.g., D-glucose contains 53% of its weight as oxygen. Chemocatalytic value addition to D-glucose may either leave its C6 skeleton intact or use C-C cleavage/formation reactions [99]. The former may involve, for example, reduction to sugar alcohols, such as sorbitol, and diols, such as isosorbide, or oxygen removal by dehydration to 5-(hydroxymethyl)furfural. Furthermore, sugars from plant biomass may be selectively oxidized to sugar acids without C-C bond cleavage or formation [100]. In this regard, the chemical synthesis of sugar acids has focused mainly on the D-glucose-based production of D-gluconic acid and D-glucaric acid. D-Glucose possesses primary and secondary alcohol and aldehyde functions that are oxidizable. Thus, aerobic selective oxidation catalysts and processes are required to obtain these acids from D-glucose in a process that maintains the 6-carbon skeleton of D-glucose intact without C-C bond formation or scission. The selective oxidation of the aldehyde group of D-glucose at position C1 to yield the carboxylic acid D-gluconic acid is well-established, while the additional oxidation of the primary hydroxyl group at position C6 is much more difficult.

The selective aerobic oxidation of D-glucose to D-gluconic acid generally is based on noble metal catalysts (Pt, Pd, Au). While Pt catalysts deactivate faster, thus, reducing D-gluconic acid yield, Pd catalysts allow high D-gluconic acid yields [100]. For example, the bimetallic Pd-Bi/C used at pH 9 with 1.66 M D-glucose at 40 °C with aeration for 2.6 h resulted in a 99.6% conversion at a catalyst ratio of 787 mol D-glucose per mol catalyst metal [101]. In comparison, an Au/TiO_2_ catalyst used at pH 11 with 0.1 M D-glucose at 40 °C with aeration for 2 h resulted in a 100% conversion at a better catalyst ratio of 4378 [102]. However, the processes suffer from leaching of the active phase limiting catalyst reutilization and from pH control by a sacrificial strong base that necessitates pH neutralization during downstream processing to isolate D-gluconic acid.

**Fig. 6:**
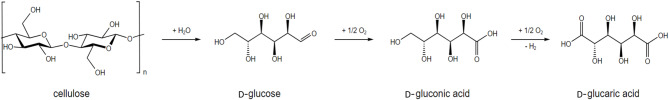
Conversion of cellulose into D-glucaric acid

Although in its infancy, direct utilization of lignocellulosics for chemocatalytic production of D-gluconic acid has been described. Starting with the disaccharide cellobiose (5.13 g/L) allowed 100% conversion with a yield of 67.4% using an Au/TiO_2_ catalyst at 120 °C with 5 bar O_2_ aeration for 3 h at the low catalyst ratio of 118 [103]. However, the price of cellobiose prevents its use for D-gluconic acid production on a large scale. To convert lignocellulosics to D-gluconic acid in a one-pot reaction, bifunctional catalysts with both acidic and metal sites are necessary for acid-catalyzed hydrolysis of lignocellulosics and selective D-glucose oxidation (Fig. 6). Using the strongly acidic heterogeneous caesium hydrogen phosphotungstate-supported Au catalyst Au/Cs_2_HPW_12_O_40_, 20 g/L cellulose were converted to D-gluconic acid during 11 h at 145 °C with 10 bar O_2_ with 60% conversion yield [104]. A transition metal catalyst based on FeCl_3_ converted cellulose to D-gluconic acid with a 50% yield [105]. This two-step process was performed at 120 °C and comprised 60% FeCl_3_ for cellulose dissolution and hydrolysis for 10 min followed by slower (110 min) oxidation in 40% FeCl_3_ hydrolysis. However, formic acid and acetic acid are major by-products [105].

Oxidation of D-glucose to the α,ω-dicarboxylic acid D-glucaric acid involves the selective oxidation of the aldehyde group at the C1 position and of the primary hydroxyl group at position C6 of D-glucose, with the latter being more difficult. This is due to the severe conditions required for the oxidation of the primary hydroxyl group that may lead to unwanted byproducts diminishing D-glucaric acid yield as a result of C-C cleavage, successive retro-aldol condensation, and D-glucose-D-fructose isomerization reactions [100]. Glucaric acid has long been produced by stoichiometric oxidation of D-glucose with nitric acid [106], with by-products D-gluconic, oxalic, tartaric, and 5-keto-gluconic acids reducing the yield to about 40%. Catalytic oxidation of D-glucose to D-glucaric acid proceeds via D-gluconic acid. In the catalytic oxidation of 3 g sodium D-gluconate to D-glucaric acid using a Pd/TiO_2_ catalyst (0.1 g, 2 wt % metal loading), 41% conversion was reached after 6 h at 60 °C in the presence of 1 g NaOH [107], but the selectivity was about 44% due to formation of by-products (28.3 tartronic acid, 8.3 oxalic acid and 11.5% for D-glyceric acid, formic acid and glycolic acid). Almost complete conversion of D-glucose to D-glucaric acid was achieved with a Pt/C catalyst at pH 9, with D-gluconic acid being formed first with an 80% yield before being oxidized further to D-glucaric acid and the final selectivity of D-glucaric acid was 57% at 97% D-glucose conversion [108].

Besides D-glucose oxidation, the selective oxidation of the hexoses D-mannose, D-rhamnose, and D-galactose as well as the pentoses L-arabinose, D-xylose, D-ribose, and D-lyxose with metallic catalysts, in particular Au, has also been described. Overall, the chemocatalytic processes for biomass conversion to sugar acids require further improvements to achieve higher carbon efficiency, faster volumetric productivity, and higher substrate loading and final product concentration before commercialization.

### Bioconversion by enzyme catalysis or whole-cell biotransformation

Enzymes typically provide excellent selectivity. Cell-free multi-enzyme catalysis allows for high conversion rates and reaction efficiencies, easy control and optimization of reaction conditions, coupled with routine product separation, while catalyst preparation (isolated enzymes, crude extracts, or whole cells) and cofactor utilization may be costly. Bioconversion of sugars to sugar acids, e.g., D-xylose to D-xylonic acid, is common. In Table 1, they are distinguished from production by fermentation and growth-decoupled production, which is described below.

Gluconic acid can be produced from D-glucose as well as from cellulose. In the latter, cellulases hydrolyze cellulose to cellobiose, which β-glucanase cleaves to D-glucose. Glucose oxidase oxidizes D-glucose to gluconolactone which is opened by spontaneous non-enzymatic hydrolysis [109]. Starch-based production of D-gluconic acid was achieved with 82% yield using glucoamylase for starch breakdown and D-glucose oxidase from *Aspergillus niger* for D-glucose oxidation immobilized non-covalently onto chemically reduced graphene oxide [110]. Glucose oxidase generates hydrogen peroxide as a stoichiometric by-product of gluconolactone and usually is detoxified by catalase. Notably, another enzyme oxidizing D-glucose to gluconolactone, namely glucose-1-dehydrogenase, is used as a coupling enzyme in cell-free biocatalysis for recycling NAD^+^ or NADP^+^ required for oxidation reactions [111].

Glucuronic acid may serve as an example of a sugar acid that can be produced by whole-cell biotransformation and enzyme catalysis using the same catalyst [112]. When *E. coli* BL21(DE3) transformed with pET28a(+) carrying the *MIOX* (*myo*-inositol oxygenase) gene from *Cryptococcus neoformans*, *myo*-inositol was converted to glucuronic acid by whole cells and two crude cell lysates (prepared either by homogenization or sonication). However, whole cells performed best (90% conversion as compared to 46% and 11%, respectively). After process optimization, the whole cells produced about 2 g/L of glucuronic acid with a conversion rate of 99% [112].

Manufacturing glucaric acid by cell-free enzyme catalysis starting with D-glucose-1-phosphate as a substrate was performed in a two-pot approach. Thermostable versions of phosphoglucomutase, *myo*-inositol-3-phosphate synthase, and inositol-1-monophosphatase converted D-glucose-1-phosphate to *myo*-inositol at 80 °C. After the vacuum concentration of samples from the first reaction, MIOX, Udh, and Nox (for NADH oxidation) yielded 3 g/L D-glucaric acid after the temperature was lowered to 40 °C. Upon immobilization of linkered versions of the enzymes (with the exception of free MIOX) onto zeolite, 1.7 g/L D-glucaric acid was produced in 10 h with 20 mol% yield [113]. With sucrose as substrate, cell-free enzyme catalysis yielded 7.3 g/L D-glucaric acid and involved cascading six enzymes: sucrose phosphorylase, phosphoglucomutase, *myo*-inositol 1-phosphate synthase, *myo*-inositol monophosphatase, MIOX, and Udh [114]. Notably, xylans may be a substrate for D-glucaric acid production. The linear homopolymer of D-xylose, xylan, occurs with branching substituents, e.g., acetylated–glucuronoxylan in hardwood, arabino-glucuronoxylan in coniferous softwood, and glucurono-arabinoxylan in cereals. These glucuronoxylans are branched with 4-*O*-methyl-glucuronic acid or glucuronic acid by α-(1→2)-linkages. Using free or scaffolded xylanase from *Flavobacterium* sp., glucuronidase from a rumen metagenomic library, and Udh from *Pseudomonas mendocina*, sub-millimolar concentrations of a mixture of D-glucaric acid and 4-*O*-methyl-D-glucaric acid was produced according to the ratio of 1,2-α-linked glucuronic acid and its 4-methyl ether present in the xylan used as substrate [115]. In a two-enzyme approach, α-glucuronidase from *Amphibacillus xylanus* debranches xylan to yield 4-*O*-methyl-D-glucaric acid, which subsequently is oxidized to 4-*O*-methyl-D-glucaric acid by gluco-oligosaccharide oxidase from *Sarocladium strictum* with a yield of 0.62 g/g 4-*O*-methyl-D-glucaric acid [116]. The remaining xylan polymer can easily be separated from the product 4-*O*-methyl-D-glucaric acid and even used for further valorization.

## Metabolic engineering for the microbial production of C5 and C6 sugar acids

Fermentative production of sugar acids starts with simple carbon and nitrogen sources and provides the advantage that the cell as the catalyst is synthesized during production. To achieve this goal, metabolic engineering alters the cell to enable efficient sugar acid production. The strategies mainly including engineering metabolic pathways to overexpress essential enzymes for sugar acid biosynthesis, deleting competing pathways to increase precursor availability and prevent product degradation, optimizing cofactor balance for efficient redox reactions, and transporter engineering to improve precursor uptake or sugar acid export are discussed in the following sections and summarized in Table 1. Cells may be engineered for growth-decoupled production, where a mixture of a carbon source supporting growth (e.g. D-glucose) and a carbon source for production (e.g. D-xylose for D-xylonic acid) is used.

**Table 1:** Production of C5 and C6 sugar acids in engineered microorganisms by fermentation and whole cell transformation (WCT). Yields are given for the respective substrates both in one carbon source growth-coupled fermentation and for growth-decoupled fermentations with carbon source mixtures

Host	Precursor uptake, catabolism, and availability	Biosynthesis	Product export and catabolism	Substrate	Conditions (Working volume-t_0_/Time/Mode)	Rounded titer (g/L)	Rounded yield (g/g)	Ref.
Production of C5 Sugar Acids								
*D-xylonic acid*								
*E. coli* W3110	*xylAB*	*xdh*_*Cc*_	*yagF*, *yjhG*	D-xylose, D-glucose	3 L/36 h/Batch	39	1 (X)	[117]
*E. coli* W3110	*xylA*, *ldhA*, *ackA*, *poxB*, *adhE*, *ptsG*, *lacZ*	*xdh*_*Cc*_, *xylC*_*Cc*_	*yagF*, *yjhG*	D-xylose, D-glucose	4 L/60 h/Fed-batch	108	1.1 (X)	[120]
				Corn cob hydrolysate (D-xylose, D-glucose, L-arabinose)	4 L/60 h/Fed-batch	91	1.1 (X)	[120]
*E. coli* BL21(DE3)	*xylAB*, cleaveble *ptsI*	*xdh*_*Cc*_, *xylC*_*Cc*_		D-xylose, D-glucose	2 L/28 h/Fed-batch	199		[121]
*C. glutamicum*	*iolR*			D-xylose, D-glucose	1.2 L/27 h/Fed-batch	36	1.1 (X)	[124]
*C. glutamicum* gX	*xylA*_*Xc*_, *xylB*_*Cg*_	*xdh*_*Cc*_		D-xylose	0.01 L/144 h/Batch	34	0.8	[126]
*C. glutamicum* ATCC 31831		*xdh*_*Cc*_		D-xylose from sawdust hydrolysate	1.5 L/168 h/Batch	49	0.9	[127]
*S. cerevisiae* B67002	*gre3*	*xdh*_*Cc*_		D-xylose, D-glucose, ethanol	0.5 L/120 h/Fed-batch	43	0.9 (X)	[130]
***L-arabinonic acid***								
*S. cerevisiae* CEN.PK 113-17A	*Gal2*	*aradh*		L-arabinose, D-glucose	0.5 L/118 h/Fed-batch	18	0.9 (A)	[131]
*E. coli* MG1655 (DE3)	*araA*	*Aradh*		L-arabinose, D-glucose	2 L/36 h/Batch	44	1.1 (A)	[132]
**Production of C6 Sugar Acids**								
***D/L-galactonic acid***								
*E. coli* BW25113	*galK*	*gld*_*Ps*_	*dgoK*	D-galactose, D-glucose	2 L/72 h/Batch	18	0.9 (Gal)	[133]
*E. coli* BW25113	*galK*	*Aradh*	*dgoK*	D-galactose, D-glucose	2 L/72 h/Batch	24		[132]
*T. reesei*			*lgd1*	D-galacturonic acid, D-xylose	0.5 L/100 h/Batch	7	0.9 (GalA)	[134]
*A. niger* ATCC 1015	*pyrG*		*gaaB*	D-galacturonic acid, D-xylose	0.5 L/171 h/ Fed-batch	5	0.9 (GalA)	[134]
***meso-galactaric acid***								
*E. coli BL21*(DE3)	*uxaC*	*udh*_*At*_	*garD*	Sugar beet (D-galacturonic acid, L-arabinose, D-glucose)	0.05 L/48 h/WCT	10	1 (GalA)	[8]
*T. reesei*	*gar1*	*udh*_*At*_		D-galacturonic acid, D-glucose	0.05 L/211 h/Batch	4	1.1 (GalA)	[139]
*A. niger* ATCC 1015 (CBS 113.46)	*gaaA*	*udh*_*At*_		D-galacturonic acid, D-glucose	0.05 L/96 h/Batch	1	0.2 (GalA)	[139]
*A. niger* ATCC 1015	*gaaA*, *gaaC*, *gaaX*	*udh*_*At*_	39114	Pectin	0.004 L/120 h/Batch	12		[144]
***D-gluconic acid***								
*K. pneumoniae* CGMCC 1.6366 (TUAC01)			*gad*	D-glucose	3 L/100 h/Fed-batch	422	1	[145]
*Aureobasidium* sp. P6		*GOD1*		D-glucose	7 L/108 h/Batch	187	1.2	[146]
*E. coli* Waksman		*pqqABCDEF*		Sucrose hydrolysate (D-glucose, D-fructose)	4 L/34 h/Batch	94	1.0 (G)	[147]
***D-glucuronic acid***								
*E. coli* BW25113		*MIOX*_*Tt*_	*uxaC*	*myo*-Inositol	12 h/WCT	106	1	[151]
***D-glucaric acid***								
*E. coli* BL21 Star (DE3)		*INO1*, *MIOX*_*Mm*_, *udh*		D-glucose	0.05 L/72 h/Batch	1	0.1	[152]
*E. coli* BL21 Star (DE3)		*INO1*, *MIOX*_*Mm*_, *udh* (Scaffolding)		D-glucose	0.05 L/48 h/Batch	3	0.3	[153]
*E. coli* MG1655 (DE3)	*uxaC*	SUMO*-MIOX*_*Mm*_, *udh*	*gudD*	*myo*-Inositol	0.05 L/72 h/Batch	5	0.5	[154]
*E. coli* BL21 (DE3)	*zwf*, *pgi*, *uxaC*	*INO1*, *suhB*, *MIOX*_*At*_, *udh*, *nox*	*gudD*	D-glucose, D-glycerol	0.1 L/72 h/Batch	5	0.5 (G)	[159]
*B. subtilis*	*pdhR, uxaC*, *yrbE*, *iolG*, *alsSD*	*INO1*, *MIOX*_*Mm*_, *udh*	*gudD*	D-glucose	0.025 L/Batch	1		[160]
*E. coli* MG1655 (DE3)	*endA, zwf*, *pfkB*, *uxaC*	*INO1*, *MIOX*_*Mm*_, *udh*	*gudD*	D-glucose	0.06 L/72 h/Batch	1	0.1	[162]
*E. coli* BL21 (DE3)	*cscB*, *cscA*, *cscK*, *zwf*, *pgi*, *glk*, *ptsG, uxaC*	*INO1*, *suhB, MIOX*, *udh*	*gudD*	Sucrose	84 h/Batch	1.4	0.14	[164]
*S. cerevisiae* BY4471	*ZWF1, opi1*, *ITR1*	*INM1*, *MIOX*_*At*_, *udh*		D-glucose, *myo*-inositol	2.5 L/168 h/Fed-batch	16	0.2(G) 1.2(MI)	[166]
*P. pastoris* GS115		*MIOX*_*Mm*_, *udh*_*Pp*_		D-glucose, *myo*-inositol	1 L/96 h/Fed-batch	7		[169]
*S. cerevisiae* BY4741	*opi1*, *ZWF1, Itr1*	*MIOX4*_*At*_, *udh*		D-glucose, *myo*-inositol	3 L/264 h/Fed-batch	11		[171]
A. *niger* ATCC1015	*oahA*, *cexA*, *zwf*, *pfkA, ScJEN1*	*udh*_*Pp*_, *MIOXA*_*An*_, *INOA*_*An*_, *nox*_*Ll*_		D-glucose, *myo*-inositol	0.05 L/120 h	0.3		[172]
*S. cerevisiae* INVSc1	*opi1*	*MIOX4*_*At*_, *udh*		D-glucose, *myo*-inositol	0.05 L/168 h/Fed-batch	10		[173]
				Avicel	0.05 L/168 h/Batch (CBP by *T. reesei* Rut-C30 and *S. cerevisiae*)	0.5	0.04	[173]
				SECS	0.05 L/168 h/Batch (CBP by *T. reesei* Rut-C30 and *S. cerevisiae*)	0.5	0.03	[173]
*S. cerevisiae* BY4741	*opi1, ras2*	*MIOX*, *udh*, *lag1*		D-glucose, *myo*-inositol	3 L/168 h/Fed-batch	10	0.2 (G)	[177]

### C5 sugar acid production

#### D-xylonic acid

**Fig. 7:**
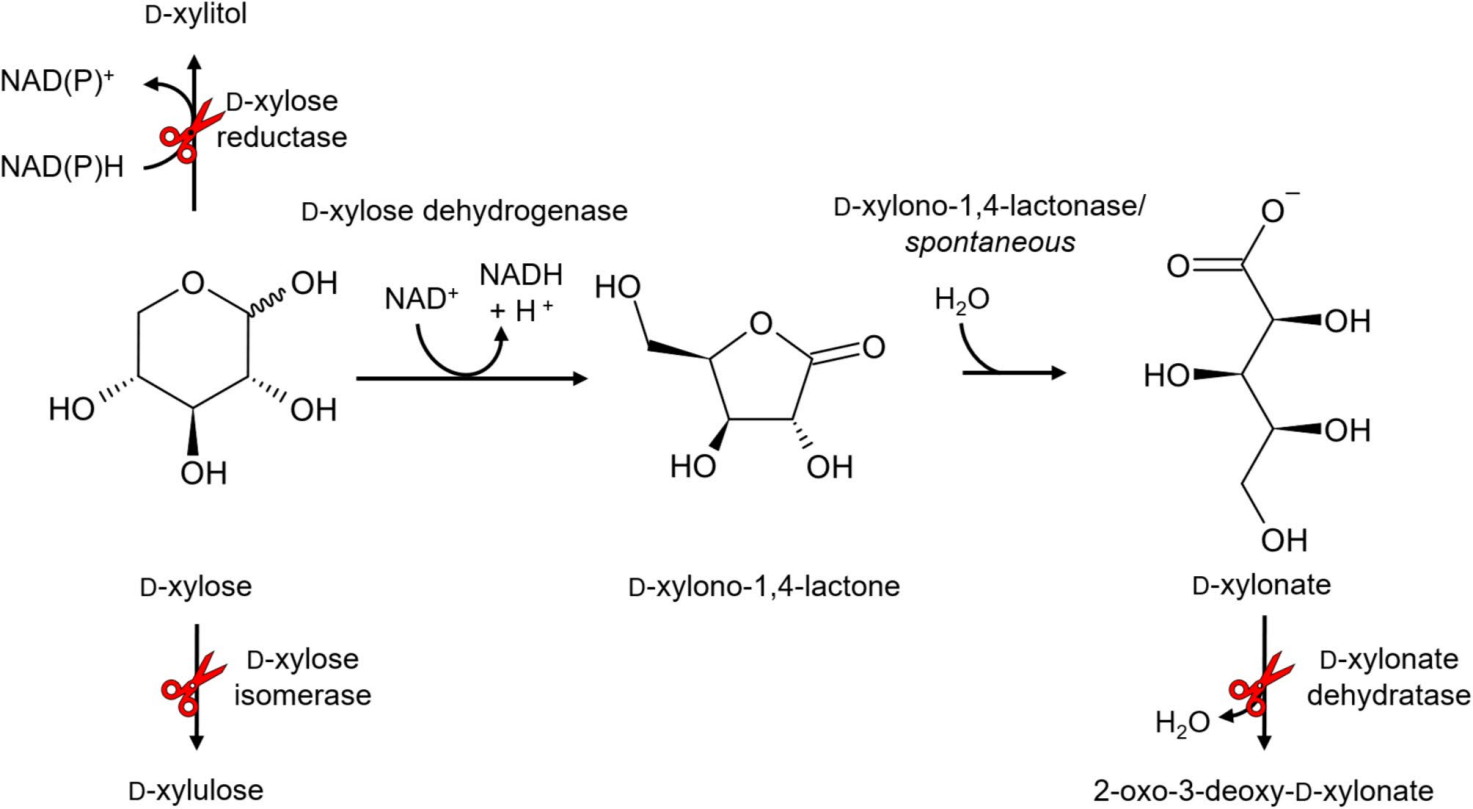
Reaction scheme of D-xylose oxidation to D-xylonic acid. Red scissors indicate catabolism genes, that were deleted in the studies to increase product titers

D-xylonic acid was produced using several metabolically engineered bacteria by feeding D-xylose. Since D-xylonic acid is an intermediate of the D-xylose catabolism, the D-xylose dehydrogenase is utilized as the main enzyme for the oxidation of D-xylose in the biosynthesis pathway. For this reason, the construction of a D-xylonic acid pathway starts with the cloning of *xdh* gene (Fig. 7). Although the next step can be performed spontaneously, XylC was shown to speed up the conversion. Precursor availability can be increased by deleting the catabolism genes, *xylA* and *xylB*. Furthermore, D-xylonic acid titers can be increased by the prevention of product consumption. Metabolic engineering studies following these strategies are summarized below.

The first bacterial production was reported by Liu et al. (2012) in *E. coli*. Since *E. coli* is natively able to utilize D-xylose and D-xylonic acid as carbon sources, the catabolic pathways had to be disabled. The D-xylose utilization operon *xylAB* consisting of the D-xylose isomerase and D-xylulose kinase genes was knocked out, resulting in a strain unable to utilize D-xylose. Additionally, *yagF* and *yjhG* encoding D-xylonate dehydratases were knocked out, leaving it unable to utilize D-xylonic acid for growth. The deletion strain was transformed with a plasmid carrying a gene for a NAD^+^-dependent D-xylose dehydrogenase *xdh*_*Cc*_ from *C. crescentus* (Note: this gene is also called *xylB*. The common name *xdh*_*Cc*_ was chosen to prevent confusion). The resulting D-xylonic acid producer strain *E. coli* W3110 Δ*xylAB* Δ*yagF* Δ*yjhG* (pET28a-*xdh*_*Cc*_) produced about 39 g/L D-xylonic acid from 40 g/L D-xylose after 36 h of incubation in 3 L M9-minimal medium, with a yield of 0.98 g D-xylonic acid/g D-xylose [117].

The formation of D-xylonic acid was improved by the expression of a D-xylonolactonase, XylC [118]. The activity of Xdh leads to the formation of D-xylono-1,4-lactone, which can then spontaneously hydrolyze to D-xylonic acid (Fig. 7). However, it was shown, that the activity of XylC_Cc_ from *C. crescentus* is up to 100-fold faster than the spontaneous reaction [55]. The strain *E. coli* BL21 Star (DE3) Δ*xylAB* was unable to utilize D-xylose and D-xylonic acid due to a lack of D-xylonate dehydratase activity in its parental strain. The derived strain *E. coli* BL21 Star (DE3) Δ*xylAB* (pACYCduet-1-*xdh*-*xylC*) produced about 27 g/L D-xylonic acid (+ 1.7 g/L lactone) from 30 g/L D-xylose after 16 h in a 5 L bioreactor. The corresponding strain without the *xylC*_*Cc*_ gene had a significantly lower growth rate. It was shown that the XylC_Cc_ activity acidified the cytoplasm decreasing the viability of cells [119].

A similar strain was used for the production of D-xylonic acid from corn cob hydrolysate [120]. In this study, XylC_Cc_ was expressed at a lower level. The hydrolysate mainly consisted of D-xylose, D-glucose, and L-arabinose. Genes for byproduct formation were knocked out to increase biomass formation and D-xylonic acid titers. Byproducts such as acetate and ethanol were almost completely abolished by the deletions. Carbon catabolite repression induced by the co-consumption with D-glucose was identified as a problem and surpassed by the knockout of *ptsG*. The final strain *E. coli* W3110 Δ*xylA* Δ*yjhG* Δ*yagF* Δ*ldhA* Δ*ackA* Δ*poxB* Δ*adhE* Δ*ptsG* Δ*lacZ* (pET*P*_*tac*_-*xdh*_*Cc*_*xylC*_*Cc*_) produced about 91 g/L D-xylonic acid from about 87 g/L D-xylose present in corn cob hydrolysate in fed-batch cultivation with an initial volume of 4 L M9-minimal medium.

Another metabolic engineering strategy circumvented the problem of carbon catabolite repression by integrating programmable biomolecular switches [121]. These switches are based on the expression of viral proteases and degradation tags. A protease-based inverter was used for fine-tuning the expression of a cleavable PtsI and Xdh_Cc_ in *E. coli* BL21(DE3) Δ*xylAB*. During growth in D-glucose and D-xylose containing medium the strain expressed first Xdh_Cc_ and cleaved PtsI protein leading to the production of D-xylonic acid, while after induction PtsI took up D-glucose and Xdh_Cc_ was cleaved, thereby effectively decoupling production from growth. An independent strategy was based on an oscillating system periodically expressing XylC_Cc_ to relieve stress from acidification in *E. coli* BL21(DE3) Δ*xylAB*. The strain had a two order of magnitude higher viability after 36 h of cultivation and twice the D-xylonic acid titer after 72 h in comparison to a control strain, that constantly expressed XylC_Cc_. When the former strain was cultivated in a 5 L fed-batch culture in TB medium, it produced about 199 g/L D-xylonic acid.

*Corynebacterium glutamicum* was chosen as an alternative host to produce d-xylonic acid. Conveniently, *C. glutamicum* is not able to natively utilize either D-xylose or D-xylonic acid as a carbon source, making it a suitable host organism. Tenhaef et al. (2018) constructed the strain *C. glutamicum* Δ*iolR* [122]. The deletion of the inositol operon repressor gene, *iolR*, leads to the derepression of several *myo*-inositol utilization genes [123] including the permease gene *iolT1*, leading to facilitated D-xylose uptake [124]. Additionally, the transcription of the *myo*-inositol 2-dehydrogenase IolG increased. This enzyme also possesses D-xylose dehydrogenase activity, catalyzing the oxidation of D-xylose to D-xylonic acid [122]. Other derepressed dehydrogenases such as OxiA might also have had an activity towards D-xylose. The strain reached the maximal theoretical yield of 1 mol D-xylonic acid/mol D-xylose in fed-batch cultivation with a final working volume of about 1.33 L in CGXII medium.

**Fig. 8:**
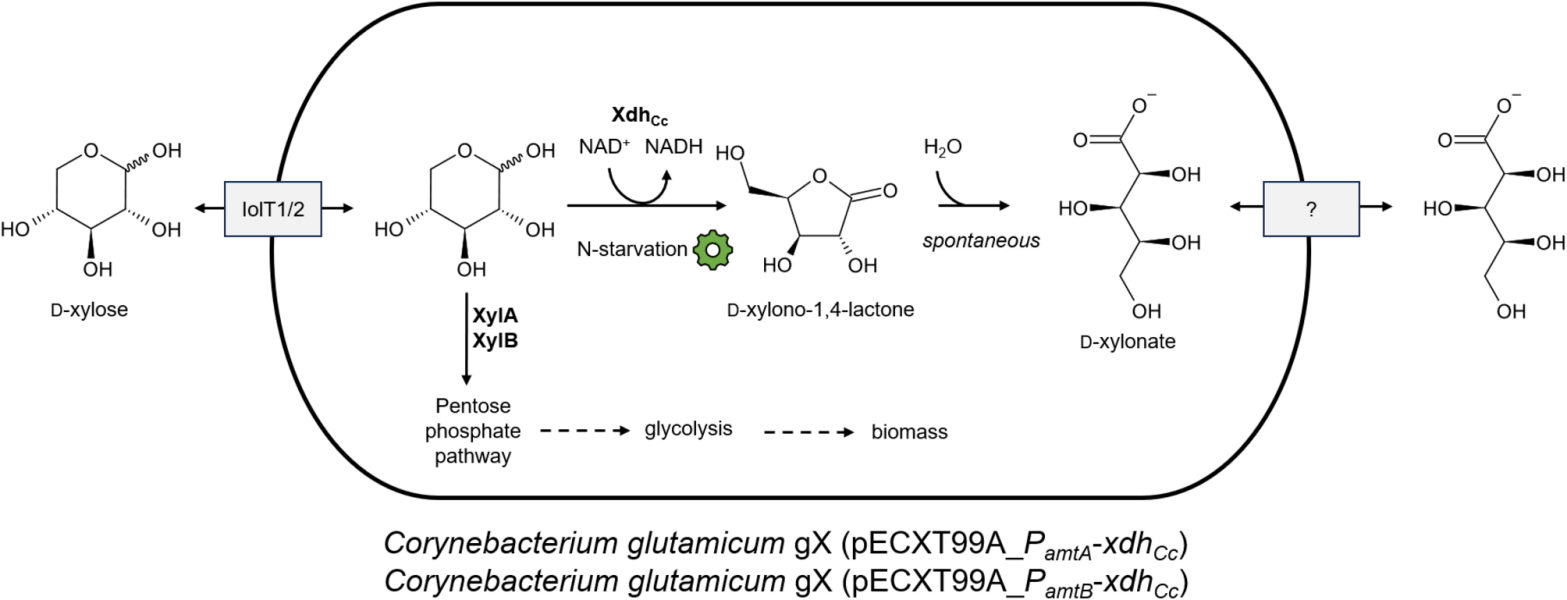
Schematic depiction of N-starvation inducible D-xylonic acid production from D-xylose by *C. glutamicum* according to [125]. The gene *xdh*_*Cg*_ was either transcribed from the endogenous nitrogen starvation-inducible promoters of the *amtA* gene or the *amtB* gene, as indicated with green gear. Overexpressed genes are represented in bold

When *C. glutamicum* gX was used as the host and a nitrogen-control circuit was installed, production increased [125]. This strain has a genomic integration of a synthetic *xylAB* operon with the *xylA*_*Xc*_ gene from *Xanthomonas campestris* and *xylB*_*Cg*_ from *C. glutamicum* that was evolved in an ALE experiment for faster D-xylose utilization for biomass [126]. To produce D-xylonic acid, different plasmids carrying the *xdh*_*Cc*_ gene under the control of different promoters were utilized. The strain *C. glutamicum* gX (pECXT99A-*xdh*_*Cc*_) (IPTG-inducible) produced 185 ± 13 mM D-xylonic acid after 72 h and a maximum of 217 ± 7 mM after 144 h, resulting in a yield of 0.8 mol D-xylonic acid/mol D-xylose in CGXII minimal medium. Further, two nitrogen (N)-starvation inducible promoters were used for the expression of *xdh*_*Cc*_ (Fig. 8). In N-CGXII medium with only 5% of the N-content of regular CGXII medium, the strains *C. glutamicum* gX (pECXT99A_*P*_*amtA*_-*xdh*_*Cc*_) and *C. glutamicum* gX (pECXT99A_*P*_*amtB*_-*xdh*_*Cc*_) produced 209 ± 7 mM and 171 ± 9 mM D-xylonic acid respectively from 267 mM D-xylose after 144 h. A significantly lower biomass accumulation was found in the N-CGXII medium than in the regular CGXII medium, thereby demonstrating the decoupling of growth and production by the N-starvation inducible promoters [125].

*C. glutamicum* ATCC 31831 was engineered to combine the expression of a pentose transporter gene with *xdh*_*Cc*_ and produced 60 g/L D-xylonic acid from sawdust hydrolysate [127]. The D-xylonic acid was purified with high purity and the possible usage of D-xylonic acid as an antimicrobial agent was assessed.

Yeasts were also engineered to produce D-xylonic acid using the same D-xylose dehydrogenase strategy as in bacteria. A *S. cerevisiae* production strain that expressed the D-xylose dehydrogenase gene *xdh*_*Tr*_ from *Trichoderma reesei* (*xyd1* in source publication) produced 1.2 ± 0.1 g/L D-xylonic acid after 53 h in fed-batch fermentation. Deleting the aldose reductase gene *gre3* reduced D-xylitol formation from D-xylose, an intermediate of D-xylose catabolism, by 67%. While the specific production remained unchanged, biomass formation was significantly reduced, resulting in lower production [128, 129]. Essentially, the same strategy was used for the alternative yeast *Kluyveromyces lactis* resulting in a production of 7.7 ± 0.4 g/L D-xylonic acid with a yield of 0.4 g D-xylonic acid/g D-xylose in batch fermentation [129]. Utilizing the D-xylose dehydrogenase gene *xylB*_*Cc*_ was superior to *xdh*_*Tr*_ in *S. cerevisiae*. Coexpression with *xylC*_*Cc*_ led to higher initial production rates [130].

#### L-arabinonic acid

Metabolic engineering studies for L-arabinonic acid production are mainly focused on the heterologous expression of the arabinose dehydrogenase (AraDH) enzyme. Aro-Kärkkäinen et al. tested four potential AraDHs from *Azospirillum brasiliense*, *Bradyrhizobium BTA1i*, *Pseudomonas fluorescens*, and *Rhizobium leguminosarum* identified through literature and bioinformatics searches to produce L-arabinonic acid from L-arabinose in *S. cerevisiae*. The most effective enzyme was found to be a D-galactose 1-dehydrogenase from *R. leguminosarum* (*Rl* AraDH). The strain expressing the *Rl* AraDH produced 4.3 g/L L-arabinonic acid at an initial production rate of 48 ± 4 mg/L/h. Upon additional overexpression of the galactose permease GAL2, 17.5 ± 1.0 g/L of L-arabinonic acid were produced at a faster rate of 248 ± 23 mg/L/h [131].

L-arabinonic acid production from L-arabinose was also achieved in *E. coli* using an AraDH from *A. brasilense* [132]. To avoid substrate competition, the *araA* gene was disrupted, creating a strain that could not grow on L-arabinose. After the batch fermentation in 2 L working volume, the strain consumed all L-arabinose within 36 h and the final titer of L-arabinonic acid was about 44 g/L with a yield of 99% (mol/mol).

### C6 sugar acid production

#### D/L-galactonic acid

**Fig. 9:**
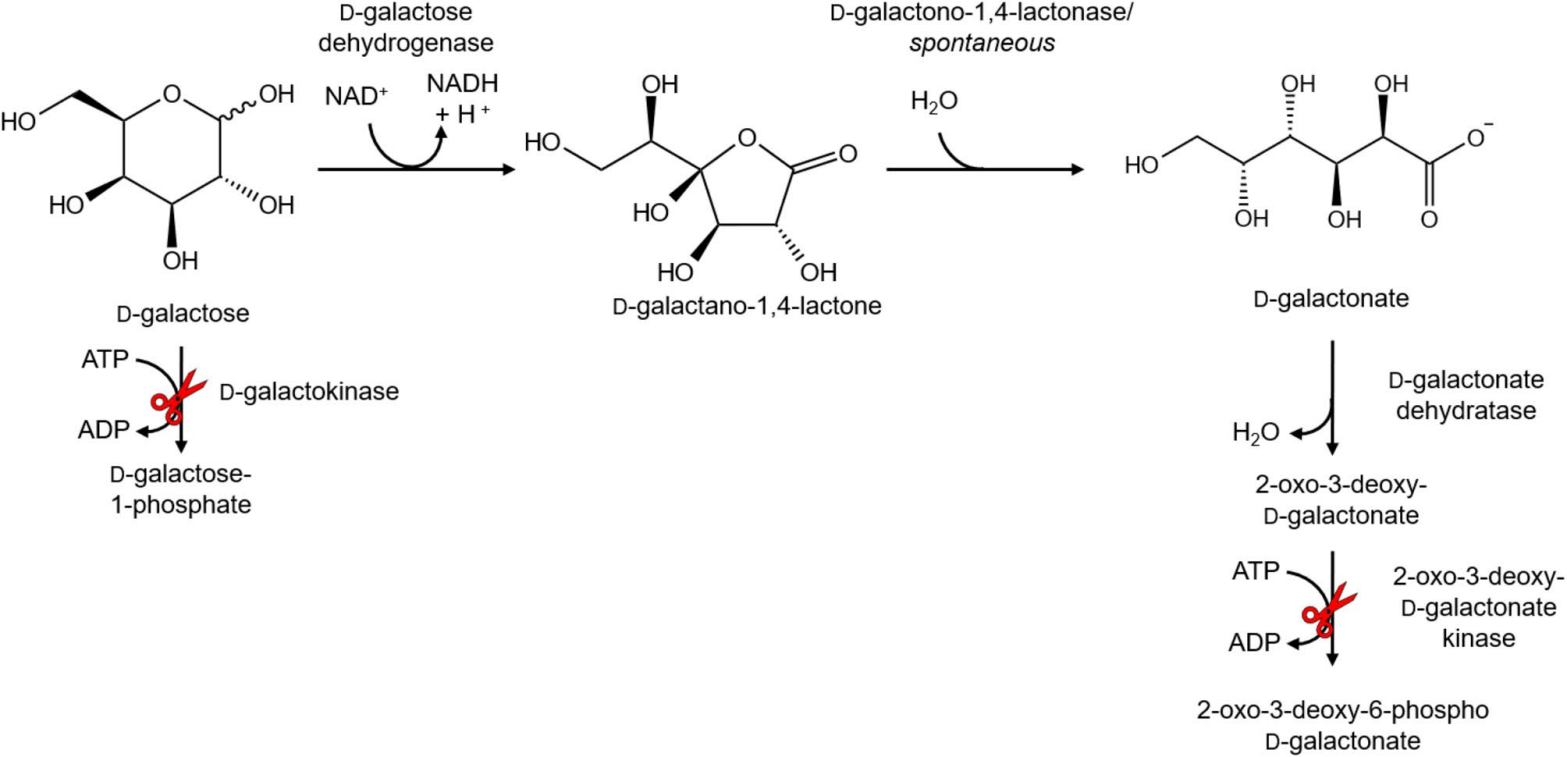
Reaction scheme of D-galactose oxidation to D-galactonic acid. Red scissors indicate catabolism genes, that were deleted in the studies to increase product titers

In *E. coli*, D-galactonic acid was produced by feeding D-galactose to a metabolically engineered strain [133]. Deleting the D-galactokinase gene *galK* and the 2-oxo-3-deoxy-galactonokinase gene *dgoK* in *E. coli* BW25113 rendered this strain unable to catabolize D-galactose and D-galactonic acid (Figs. 4 and 9). Expression of D-galactose dehydrogenase gene *gld*_*Ps*_ from *P. syringae* led to 18 g/L D-galactonic acid whereas L-arabinose dehydrogenase from *A. brasilense* that had a higher catalytic efficiency towards D-galactose, improved the titer by 36% to 24 g/L [132].

*T. reesei* and *A. niger* were engineered to produce L-galactonic acid from D-galacturonic acid [134]. Since L-galactonic acid is an intermediate in the fungal D-galacturonic acid degradation, a single deletion of the respective L-galactonic acid dehydratase genes *lgd1*_*Tr*_ and *gaaB*_*An*_ was sufficient due to endogenous D-galacturonate reductase activity. The growth of the resulting strains was drastically reduced on D-galacturonic acid as the sole carbon source. *T. reesei* Δ*lgd1* produced about 7 g/L L-galactonic acid in a bioreactor in 500 mL medium with yields up to 0.85 g L-galactonic acid/g D-galacturonic acid after 100 h. *A. niger* Δ*gaaB* produced about 5 g/L L-galactonic acid with a yield of 0.9 g L-galactonic acid/g D-galacturonic acid after 171 h. The strain produced L-galactonic acid with a similar yield of 0.85 g/g when fed with polygalacturonate. Additional overexpression of the D-galacturonic acid reductase GaaA in *A. niger* increased the production rate significantly.

In *S. cerevisiae* [51, 135, 136], a D-galacturonate reductase gene from *Cryptococcus diffluens* was introduced. Since *S. cerevisiae* is natively not able to metabolize D-galacturonic acid or L-galactonic acid, the resulting strain could efficiently produce L-galactonic acid. A conversion rate of about 96% was achieved [135]. A similar strain was used for the production of L-galactonic acid from sugar beet press pulp hydrolysate [136].

#### Galactaric acid

Since galactaric acid (also known as mucic acid) is a meso-dicarboxylic acid, it is an attractive product for metabolic engineering efforts. Hitherto, galactaric acid was only produced by bioconversion of D-galacturonic acid, utilizing uronate dehydrogenase, the initial step of D-galacturonic acid catabolism. D-galacturonic acid can be hydrolyzed from the renewable polymer pectin [137, 138].

A combined approach of metabolic engineering, enzymatic conversion, and chemical synthesis to produce galactaric acid and convert it further to adipic acid was followed. Crude extract of *E. coli* BL21(DE3) (pET46-*udh*_*At*_) cells harboring a heterologously expressed uronate dehydrogenase from *A. tumefaciens* oxidized D-galacturonic acid to galactaric acid (Fig. 5) [8]. Deletion of *uxaC*, the first step of D-galacturonic acid degradation in the Ashwell isomerase pathway, and *garD*, the first step of galactaric acid degradation, increased the yield. Biocatalysis with the *E. coli* BL21(DE3) Δ*uxaC* Δ*garD* (pET46-*udh*_*At*_) strain led to a conversion rate of about 95%. As an application example, D-galacturonic acid present in enzymatically prepared sugar beet hydrolysate was used to produce galactaric acid, which was then chemically converted in a two-step reaction to adipic acid with an overall yield of 8.4%. The yield was limited by the inefficient release of D-galacturonic acid from the sugar beet pectin and the lower purity in comparison to commercially available D-galacturonic acid [8].

Most metabolic engineering studies for the production of galactaric acid focus on microbial fungi, following essentially the same strategies as described above for *E. coli* [139]. Upon transformation of a D-galacturonate reductase deficient *T. reesei* strain with the *udh*_*At*_ gene, the strain regained the ability to grow on D-galacturonic acid, albeit slower than the wild type, indicating a D-galacturonic acid-independent galactaric acid utilization pathway. This *T. reesei* strain produced 3.8 ± 0.1 g/L galactaric acid from 17.4 g/L D-galacturonic acid after 211 h in shake flasks containing pure D-galacturonic acid solution at pH 5.5. The yield matched the highest theoretically possible yield with 1.08 ± 0.04 g galactaric acid/g D-galacturonic acid. The overall production was the highest (5.9 g/L) when cells were incubated at pH 6.5. For *A. niger* 1.0 ± 0.0 g/L galactaric acid with a yield of 0.16 g galactaric acid/g D-galacturonic acid were produced in 4 days. The authors proposed that the export of galactaric acid might be limiting in *T. reesei*, where further engineering could prove beneficial [139]. The culture conditions for the *T. reesei* strain were later optimized and led to a final production of about 20 g/L in a 1 L fed-batch cultivation with additional feeding of lactose, ammonium, and yeast extract at pH 4 and 35 °C [140]. The cultivation was carried out at different scales from 4 mL in a 24-well plate to a 250 L bioreactor [141]. In the 250 L culture, 2.8 kg galactaric acid were produced with a yield of 0.77 g galactaric acid/g D-galacturonic acid. The used D-galacturonic acid was enzymatically hydrolyzed from pectin since *T. reesei* has only limited pectinase expression [142]. A promoter exchange for the expression of *udh*_*At*_ led to significantly higher production with D-galacturonic acid as substrate but did not positively affect the production from hydrolyzed pectin [141].

*A. niger* possesses a more efficient ability to break down pectin than *T. reesei* [143]. *A. niger* cells were cultivated on galactaric acid for RNA-Seq analysis. Upregulated candidate genes, that might be involved in the catabolism were identified and a CRISPR/Cas9 strategy was used to create deletion mutants. Three out of seven mutants revealed completely abolished or reduced galactaric acid catabolism. For the strain with the deletion of the gene 39114 encoding an AMP-dependent synthetase and ligase, the consumption was fully abolished. Consequentially, the strain *A. niger* Δ*pyrG* Δ*gaaA::pyrG* Δ*39114::pyrG udh*_*At*_ was able to convert almost all of the fed D-galacturonic acid to galactaric acid while the strain without the deletion of the gene 39,114 only converted only about 7%. The strain produced about 30% of the theoretical maximum yield of galactaric acid from pectin-rich biomass. Additional deletions of *gaaX* encoding a repressor for pectin degradation and the L-galactonate dehydratase gene *gaaC*, involved in D-galacturonic acid catabolism led to further improvements [144].

A D-galacturonic acid transporter was identified in *Neurospora crassa* and cloned into *S. cerevisiae* [51]. Combined with overexpression of *udh*_*A*t_, this resulted in increased uptake of D-galacturonic acid and subsequent conversion to galactaric acid. The transporter was also beneficial for L-galactonic acid production.

#### D-gluconic acid

Although D-gluconic acid is produced in high yields by using wild-type strains, several microorganisms were engineered for D-gluconic acid production. Although metabolic engineering studies were generally performed to produce 2-oxo-L-gluconic acid or 5-oxo-D-gluconic acid, here, the studies that focus on the production of D-gluconic acid using metabolic engineering are summarized.

D-gluconic acid is an intermediate in the D-glucose oxidation pathway of *Klebsiella pneumoniae* with 2,3-butanediol as the main metabolite. By deleting the *gad* gene, which eliminates D-gluconate dehydrogenase activity, D-gluconic acid accumulated in the culture broth. During fed-batch fermentation, the engineered *K. pneumoniae* Δ*gad* strain produced a final D-gluconic acid concentration of 422 g/L, with a D-glucose-to-D-gluconic acid conversion ratio of 1 g/g [145].

To enhance the production of Ca^2+^-D-gluconic acid in *Aureobasidium* sp. P6, the glucose oxidase gene (*GOD1*) of *Aureobasidium* sp. P6 was deleted and overexpressed in the strain. Deletion of the *GOD1* gene resulted in a loss of GOD1 activity and D-gluconic acid production, while its overexpression boosted Ca²⁺-D-gluconic acid yield (161 g/L) and GOD1 activity (1.5 U/g of protein) compared to the parent P6 strain (19 g/L, 1.1 U/g of protein). During a 10 L fermentation, the overexpressing strain grown in a medium with 160 g/L of D-glucose produced about 187 g/L of Ca^2+^-D-gluconic acid, with a yield of 1.2 g/g of D-glucose and a volumetric productivity of 1.7 g/L/h [146].

A preliminary study (data not published) discovered that wild *E. coli* Waksman (*E. coli* W) strains were capable of synthesizing the apo-glucose dehydrogenase (apo-GDH) for D-gluconic acid production but lacked pyrroloquinoline quinone (PQQ) to activate it [147]. It has been suggested that the addition of external PQQ can convert apo-GDH enzyme into its holo-GDH form [148, 149]. To achieve PQQ biosynthesis, the *pqqABCDEF* operon from *K. pneumoniae* was transferred into *E. coli* W [150]. By using D-glucose from sucrose hydrolysis and optimizing the medium components with a central composite design, the recombinant strain produced up to 94 g/L D-gluconic acid from 95 g/L D-glucose [147].

#### D-glucuronic acid

**Fig. 10:**
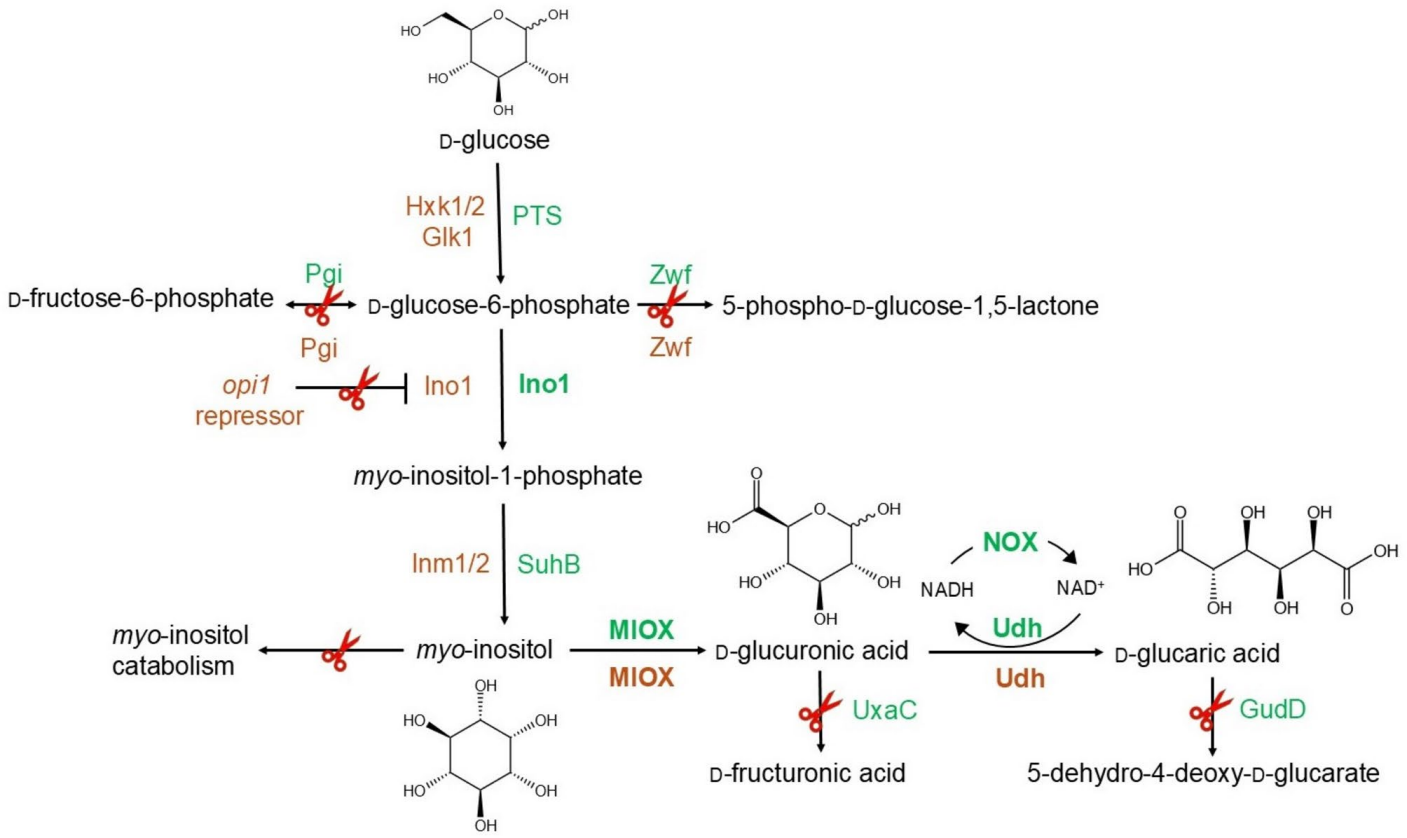
Construction of a D-glucaric acid production pathway in *E. coli* and *S. cerevisiae* using metabolic engineering. Pathways of *E. coli* and *S. cerevisiae* are given in green and orange, respectively. Heterologously expressed proteins are indicated in bold for both organisms. Scissors indicate deletions

Glucuronic acid can be produced from *myo*-inositol through one-step biocatalysis by MIOX in the presence of oxygen. Five MIOX-encoding genes from *C. neoformans*, *Chaetomium thermophilum*, *Arabidopsis thaliana*, *Thermothelomyces thermophila*, and *Mus musculus* were overexpressed in *E. coli* BW25113, with the MIOX from *T. thermophila* (TtMIOX) demonstrating high specific activity (5.1 U/mg) and converting *myo*-inositol to D-glucuronic acid efficiently. Due to the inherent instability of MIOX in vitro, a whole-cell biocatalyst expressing MIOX was employed and the *uxaC* gene was inactivated in the *E. coli* genome to prevent the product catabolism (Figs. 5 and 10). The resulting strain produced about 106 g/L of D-glucuronic acid with a conversion rate of 91% and 8.83 g/L/h volumetric productivity [151].

#### D-glucaric acid

The biosynthesis of D-glucaric acid in microorganisms can be achieved through the heterologous expression of specific enzymes (Fig. 10), utilizing D-glucose or *myo*-inositol as precursor molecules. D-glucose undergoes a two-step conversion into *myo*-inositol via the expression of either endogenous or heterologous enzymes. The next step, the production of glucuronic acid from *myo*-inositol, represents the rate-limiting step in D-glucaric acid biosynthesis due to the inherently low activity and stability of MIOX. The final step in the biosynthetic pathway involves the oxidation of D-glucuronic acid to D-glucaric acid, catalyzed by Udh derived from various organisms. To facilitate this conversion, NAD^+^ regeneration can be enhanced through the heterologous expression of Nox. Given that glucuronic acid serves as a direct precursor to D-glucaric acid, its intracellular concentration can be elevated by deleting the *uxaC* gene, which encodes a key enzyme in its catabolic pathway (Figs. 5 and 10). Additionally, D-glucaric acid yield can be further improved by disrupting the *gudD* gene, which prevents its conversion into 5-dehydro-4-deoxy-D-glucaric acid.

Fermentative production of D-glucaric acid was first demonstrated in *E. coli* through the expression of three heterologous enzymes, *myo*-inositol-1-phosphate synthase (INO1) from *S. cerevisiae*, MIOX from *M. musculus*, and Udh from *P. syringae* [152]. About 1 g/L of D-glucaric acid was produced from 10 g/L of D-glucose.

Several challenges had emerged regarding biosynthesis including the low catalytic activity and instability of MIOX, by-product formation during the overflow of carbon flux, and the competition between cell growth and D-glucaric acid production. Further studies mainly focused on overcoming these challenges using different strategies and chassis strains (Fig. 10). Here, the strategies are presented for the bacteria first, and then the yeast/fungi.

To overcome limitations caused by MIOX, different strategies were followed. An in vivo scaffold with specific ligands that combined the functional domains of INO1, Udh, and MIOX was developed to enable enzyme colocalization, significantly enhancing MIOX activity. This approach increased D-glucaric acid production from D-glucose to 2.5 g/L with a yield of 0.25 g/g, which is significantly higher than the production without the scaffold in *E. coli* [153]. Protein fusion tags were also investigated to enhance MIOX solubility. An N-terminal Small Ubiquitin-like Modifier (SUMO) fusion with MIOX led to a 75% increase in D-glucaric acid production from *myo*-inositol. A 941 bp DNA fragment that, when expressed, enhanced *myo*-inositol transport was identified and overexpression of PtsG resulted in a 65% increase in D-glucaric acid production from *myo*-inositol. Overall, the production of D-glucaric acid up to about 5 g/L from about 11 g/L *myo*-inositol in recombinant *E. coli* was achieved [154].

Ding et al. [155] randomly mutated the *MIOX* gene and screened the high-titer D-glucaric acid producer strains with an in vivo transcription factor-based D-glucaric acid biosensor (using transcription factor CdaR) and tetracycline efflux pump protein TetA. The stability and activity of MIOX were enhanced by fusing it with a SUMO and a titer of about 5.5 g/L was achieved in *E. coli*, a 17-fold improvement over the original strain. Another mutant library of SUMO-MIOX fragments was screened using a one-pot two-strain system based on a D-glucaric acid biosensor system. The D82Y and S173N MIOX mutants exhibited approximately 3.8- and 2.7-fold higher activity towards *myo*-inositol compared to the wild-type [156].

A dynamic control method [157] was also reported to regulate the expression of MIOX in *E. coli* to prevent catalytic activity lost over time. By using *myo*-inositol-responsive promoter to control MIOX expression, the D-glucaric acid titer from D-glucose increased 2.5-fold compared to the unregulated MIOX control. The implementation of a regulation for dynamically switching cells from growth to production mode resulted in a final D-glucaric acid titer of about 2 g/L, corresponding to a 20% yield by mass.

To prevent the byproduct formation and to direct the carbon flow to D-glucaric acid biosynthesis, several strains were constructed. *E. coli* MG1655(DE3) Δ*endA* Δ*recA* Δ*pgi* Δ*zwf* Δ*uxaC* Δ*gudD* strain was generated to consume alternative carbon sources and to prevent the degradation of D-glucuronic acid and D-glucaric acid. D-glucaric acid pathway was constructed by expressing SUMO-MIOX, INO, and Udh proteins. This strain demonstrated the ability to consume L-arabinose, D-glycerol, and D-xylose even in the presence of D-glucose, with D-glucaric acid yields on D-glucose increasing by 9- to 18-fold in the *Δpgi Δzwf* strain [158]. About 1 g/L D-glucaric acid was produced with a yield of about 0.7 g/g on D-glucose when D-xylose was used as the carbon source. In another *E. coli Δpgi Δzwf* strain, the D-glucaric acid pathway was constructed by introducing the *suhB*-*INO1*-*MIOX*-*udh* genes. *uxaC* and *gudD* were deleted to block the conversion pathways of D-glucuronic acid and D-glucaric acid into by-products. An in situ NAD^+^ regeneration system was introduced via NADH oxygenase. The activity of the key enzyme, MIOX, was fine-tuned by using different RBSs. About 5 g/L D-glucaric acid production with a maximized yield of about 46 mol% on D-glucose and D-glycerol by batch fermentation was achieved [159].

The D-glucaric acid production pathway was also introduced into *Bacillus subtilis* by integrating *INO1*, *MIOX*, and *udh* into the genome. Four genes (*uxaC*, *gudD*, *yrbE*, and *iolG*) were deleted to maximize the accumulation of D-glucaric acid. The native promoter of the gene *suhB* was replaced by the promoter P43. The resulting strain was able to produce about 0.2 g/L of D-glucaric acid. The construction of a feedback loop that relies on pyruvate increased the titer of D-glucaric acid to about 0.5 g/L. By blocking the by-product, acetoin, formation, the titer of D-glucaric acid reached about 0.8 g/L [160].

For the dynamic control of growth and production, dynamic growth/production switching for D-glucaric acid production was developed in *E. coli*. A degradation tag called SsrA was added to the coding sequence of Pfk-1. This allowed for the regulation of Pfk-1’s half-life by inducing the expression of SspB, which facilitated the degradation of SsrA-tagged proteins by binding to both ClpXP (the protein degradation machinery) and the SsrA tag itself. When the desired growth density was reached, Pfk-1 levels could be reduced by inducing SspB, thereby redirecting carbon flux into the heterologous pathway for D-glucaric acid production. This adjustment led to the accumulation of D-glucose-6-phosphate and resulted in a 42% increase in D-glucaric acid production compared to strains without this system [161].

A quorum-sensing based circuit was applied to *myo*-inositol and D-glucaric acid production in *E. coli*, which can be produced from D-glucose-6-phosphate by one or three heterologous enzymatic reactions, respectively. Expression of Pfk-1 was downregulated in response to cell density, enabling metabolic flux redirection to the heterologous pathway and a switch from growth to D-glucaric acid production. Approximately, a 5.5-fold increase was observed in the *myo*-inositol titer (1.28 g/L), while 0.85 g/L D-glucaric acid was produced [162].

Hou et al. developed a dynamic turn-off switch (dTFS) and a dynamic turn-on switch (dTNS) using growth phase-dependent promoters and degrons to uncouple cell growth from D-glucaric acid biosynthesis. *E. coli* MG1655 was used as the host in which the *uxaC*,* gudD*, and *pfkA* genes were knocked out. The pathway enzymes MIOX from *M. musculus* and Udh from *P. syringae* were overexpressed. Besides, INO1 from *S. cerevisiae* was inserted into pre-established dTNSs, and 6-phosphofructokinase I encoded by *pfkA* was inserted into the dTFSs. In this way, D-glucaric acid production was increased up to 1.56 g/L in a 5 L fermenter [163].

Several bacterial strains were also constructed to utilize alternative substrates for D-glucaric acid production. A novel pathway from sucrose to D-glucaric acid was developed by co-expressing *cscB*, *cscA*, *cscK*, *INO1*, *MIOX*, *udh*, and *suhB* in *E. coli* BL21(DE3). Additionally, by deleting the chromosomal genes *zwf*, *pgi*, *ptsG*, *uxaC*, and *gudD*, overexpressing *glk*, and implementing a D-fructose-dependent translation control system for *pgi*, the strain was enabled to utilize sucrose as the sole carbon source while achieving high product titer and yield. D-Fructose from sucrose was used for cell growth, and the D-glucose from sucrose flowed into the D-glucaric acid pathway. In M9 medium containing 10 g/L sucrose, the D-glucaric acid titer reached approximately 1.4 g/L, with a yield of about 14 weight-% sucrose [164].

Similar strategies applied for bacteria were also used in yeast and fungi for glucaric acid production.

The *MIOX4* gene from *A. thaliana* and the *udh* gene from *P. syringae* were integrated into the delta sequence of the *S. cerevisiae* genome to increase both the number of target gene copies and their stabilities. D-glucaric acid titer was increased to 6 g/L using 20 g/L D-glucose and 10.8 g/L *myo*-inositol in a fed-batch fermentation in a 5 L bioreactor [165].

To enhance both MIOX stability and activity, MIOX4 and Udh enzymes were fused with a (EA3K)_3_ peptide linker and D-glucaric acid production was increased up to 5.7-fold in comparison to free enzymes [166]. Integration into delta sequence sites of the *S. cerevisiae opi1* mutant, high-throughput screening with an *E. coli* D-glucaric acid biosensor strain, and downregulating *ZWF1* while overexpressing *INM1* and *ITR1*, increased D-glucaric acid production to 8.5 g/L in the final strain during shake flask fermentation. Ultimately, in a 5 L bioreactor, about 16 g/L D-glucaric acid was produced through fed-batch fermentation.

Cheah et al. [167] explored intracellular compartmentalization in *S. cerevisiae* by incorporating Murine polyomavirus virus-like particles (MPyV VLPs) for MIOX. Encapsulation of MIOX within self-assembled MPyV VLPs in yeast resulted in a 20% higher D-glucaric acid titer.

Several homologs and mutants of MIOX were screened to increase the activity. In a screen with MIOX homologs [168], D-glucaric acid production was observed with 31 enzymes, 25 of which were characterized for the first time, in *S. cerevisiae*. Expression of FjMiox (Miox from *Flavobacterium johnsoniae*) and TmMIOX (MIOX from *Talaromyces marneffei*) resulted in the highest D-glucaric acid titers (1.85 ± 0.10 and 1.76 ± 0.33 g/L, respectively) and volumetric productivity (0.019 ± 0.001 and 0.018 ± 0.003 g/L/h) from 20 g/L D-glucose and 10 g/L *myo*-inositol among the tested variants.

Identification of an endogenous PpMIOX as a functional enzyme led to the first construction of a D-glucaric acid biosynthetic pathway in *Pichia pastoris*. Co-expressing the native PpMIOX with Udh from *Pseudomonas putida* KT2440 resulted in a noticeable accumulation of D-glucaric acid (about 0.09 g/L) from *myo*-inositol. Co-expressing heterologous mouse MIOX (MmMIOX) and Udh yielded higher D-glucaric acid titers. Implementing a fusion expression strategy with flexible peptides significantly increased the specific activity of MmMIOX and the concentration of D-glucaric acid to about 7 g/L using D-glucose and *myo*-inositol as carbon sources in fed-batch cultures [169].

To reduce the flux of D-glucose towards biomass and increase the yield of D-glucaric acid, a four-step D-glucaric acid pathway (*INO1*-*INM1*-*MIOX*-*udh*) was introduced into a Pgi1p-deficient *S. cerevisiae* strain [170]. Since high D-glucose concentrations are toxic to Pgi1p-deficient strains, various feeding strategies and the use of polymeric substrates were explored. The conversion of D-glucose to D-glucaric acid was confirmed using uniformly labeled D-[^13^C]-glucose. In batch bioreactor cultures with pulsed D-fructose and ethanol supplementation, 1.3 g/L of D-glucaric acid was produced.

The effects of overexpressing the *myo*-inositol transporter *Itr1*, expressing a fusion of MIOX4 and Udh, and downregulating the glucose-6-phosphate dehydrogenase gene *ZWF1* on D-glucaric acid production were explored in *S. cerevisiae*. The results indicated that overexpressing *Itr1* led to a 26% increase in D-glucaric acid yield compared to the original strain in shake flask fermentation. Expressing the MIOX4-Udh fusion protein further boosted D-glucaric acid yield by 40%. D-Glucaric acid production reached 5.5 g/L, representing a 60% increase. In a 5 L fermenter, an 80% increase was obtained with the maximum D-glucaric acid titer of almost 11 g/L [171].

*A. niger* was also used as the host for D-glucaric acid production [172]. By expressing the uronate dehydrogenase gene (*udh*_*Pp*_) from *P. putida* KT2440 a titer of about 0.02 g/L was achieved. The overexpression of the endogenous inositol oxygenase (MIOXA_An_) and inositol-1-phosphate synthase (INOA_An_), along with the carboxylate transporter (scJEN1) from *S. cerevisiae* S288C, significantly improved D-glucaric acid production, reaching a titer of 0.1 g/L. By establishing an NAD^+^ cofactor recycling system through the expression of NADH oxidase (nox_Ll_) from *L. lactis* subsp. *cremoris* MG1363 further enhanced the D-glucaric acid yield to 115.65 mg/L. Finally, reducing the carbon flux towards glycolysis and the pentose phosphate pathway enabled the highest D-glucaric acid production of 0.3 g/L.

Cellulose utilization for D-glucaric acid biosynthesis was accomplished using a microbial consortium system that included *T. reesei*, a cellulose-degrading fungi. The D-glucaric acid biosynthesis pathway was constructed in *S. cerevisiae* INVSc1 *Δopi1*. The preferred LGA-1 strain produced almost 10 g/L D-glucaric acid from 30 g/L D-glucose and 10.8 g/L *myo*-inositol in fed-batch fermentation mode. Consolidated bioprocessing (CBP) using an artificial microbial consortium composed of *T. reesei* Rut-C30 and *S. cerevisiae* LGA-1 resulted in a D-glucaric acid titer of 0.54 ± 0.12 g/L from 15 g/L Avicel and 0.45 ± 0.06 g/L D-glucaric acid from 15 g/L steam-exploded corn stover (SECS) after 7 d of fermentation [173]. Later on, *T. reesei* was engineered for enhanced cellulase production and improved production of fermentable sugars from lignocellulose. Then, *S. cerevisiae* was genetically modified to confer its capability of cellobiose metabolism and improve the efficiency of D-glucaric acid biosynthetic pathway. The cellodextrin transport system from *N. crassa* was cloned into *S. cerevisiae* and a scaffolding strategy was applied for the enzymes in the D-glucaric acid production pathway. The titer, yield, and productivity of D-glucaric acid produced from 50 g/L SECS by the microbial consortium of *T. reesei* and *S. cerevisiae* were 6.42 g/L, 0.128 g/g SECS, and 0.917 g/L/d, respectively [174]. D-glucaric acid production was increased up to almost 12 g/L in the shake flask and almost 16 g/L in the 10 L airlift fermenter by the same consortia using different lignocellulosic substrates with different pretreatment strategies [175].

It was reported that overexpressing MIOX increases the production of D-glucaric acid but also leads to the generation of reactive oxygen species (ROS) that affect microbial cell viability [176]. Microbial cell viability was improved by reducing ROS accumulation through second codon engineering to fine-tune ceramide synthase (*lag1*) in *S. cerevisiae*, resulting in D-glucaric acid production reaching 9.5 g/L with a productivity of 0.057 g/L/h [177].

## Conclusions, challenges, and future perspectives

Microbial production of sugar acids can contribute significantly to the shift toward a bio-based economy, supporting environmental sustainability. By taking a holistic approach that combines strain engineering, process optimization, and sustainability assessments, microbial sugar acid production can emerge as a competitive and environmentally friendly alternative in the bio-based economy.

Methodology push will accelerate strain development for sugar acid production, e.g., by CRISPR technologies [178]. Genome editing by gene deletion [179] or base editing [180] can be facilitated by various CRISPR systems. Gene repression by CRISPR interference can be used for metabolic engineering [181] and parallel testing of gene targets in screening applications [182, 183]. Gene activation by CRISPRa, likewise, accelerates gene target identification [184]. To overcome toxicity problems of by-products or products, ALE that operates on the cell level with cell growth as readout, will be conducive. Strains that grow faster in the presence of a non-native substrate such as D-xylose [126] or in the presence of an inhibitor such as methanol [185] can easily be selected. The ALE approach has been extended to include the evolution of consortia [186] and product traits after flux enforcement [187, 188] or by using biosensors [189–191]. However, to deduce broadly applicable metabolic engineering strategies, it is important to identify causal mutations by genome sequencing and genetic testing. Rate-limiting enzymes such as MIOX may be improved either since more gene and amino acid sequences as well as three-dimensional structure prediction (e.g., AlphaFold3) allow us to access natural diversity [192], while on the other hand, enzyme evolution [193] enables us to even select for new-to-nature catalytic potential as shown for new-to-nature (bio)synthesis [194, 195]. These enzymes may either be fused or scaffolded to orchestrate their action [196–198].

The sustainable production of sugar acids faces several challenges. There are and will be economic requirements (e.g., petrol oil and sugar pricing) and regulatory constraints (e.g., competing uses of substrates for human nutrition). Process-inherent features such as downstream processing to purify the target sugar acid from culture supernatants that contain by-products, residual components of the biomass hydrolysates used as feedstocks as well as potentially inhibiting or toxic compounds arising from cellular conversion of feedstock components are significant challenges. We consider the stability and activity of the key biosynthetic enzyme a pivotal challenge while enhancing precursor and cofactor supply and blocking pathways leading to by-products is largely established with very good metabolic engineering strategies available. Transport engineering is more demanding since much less is known about these systems or strategies to change their substrate scope. Engineering access to the substrates present in complex hydrolysates employed in sustainable sugar acid production can be considered established for most biomass hydrolysates. Toxicity issues remain. Most hydrolysates that contain few inhibitors are dilute, but high substrate loading is required for high titer, yield, and rate bioprocesses. The choice of the production host and/or strain development by ALE are the likely successful strategies to overcome the toxicity issue. Utilizing hydrolysates offers several benefits. The co-utilization of available carbon sources enables growth-decoupled production of sugar acids. It is more cost-effective to use inexpensive hydrolysates for microbial growth than in chemical synthesis. Depending on the organism, certain carbon sources can be selectively used for cell growth, while others can be oxidized to their respective acids. As a result, tedious purification steps are not required, as separating chemically similar sugars, especially epimers, can be challenging. Overall, we believe that sustainable sugar acid production can become a success story of biotechnology.

## Data Availability

No datasets were generated or analysed during the current study.

## Abbreviations

ALE: Adaptive laboratory evolution
AraDH: Arabinose dehydrogenase
CBP: Consolidated bioprocessing
IT: Ion Transporter Superfamily
MFS: Major Facilitator Superfamily
MIOX: myo-Inositol oxygenase
MPyV VLPs: Murine polyomavirus virus-like particles
PQQ: Pyrroloquinoline quinone
RBS: Ribosome binding site
ROS: Reactive oxygen species
SBP: Solute binding protein
SUMO: Small ubiquitin-like modifier
TRAP: Tripartite ATP-independent periplasmic transporters
Udh: Uronate dehydrogenase
WCT: Whole cell transformation
